# Novel Highly Pathogenic Avian Influenza A(H5N6) Virus in the Netherlands, December 2017

**DOI:** 10.3201/eid2404.172124

**Published:** 2018-04

**Authors:** Nancy Beerens, Guus Koch, Rene Heutink, Frank Harders, D.P. Edwin Vries, Cynthia Ho, Alex Bossers, Armin Elbers

**Affiliations:** Wageningen Bioveterinary Research, Lelystad, the Netherlands (N. Beerens, G. Koch, R. Heutink, F. Harders, C. Ho, A. Bossers, A. Elbers);; Netherlands Food and Consumer Product Safety Authority, Utrecht, the Netherlands (D.P.E. Vries)

**Keywords:** HPAI, genome sequence, reassortant, H5N6, ducks, wild birds, clinical signs, influenza, viruses, highly pathogenic avian influenza, the Netherlands, respiratory infections

## Abstract

A novel highly pathogenic avian influenza A(H5N6) virus affecting wild birds and commercial poultry was detected in the Netherlands in December 2017. Phylogenetic analysis demonstrated that the virus is a reassortant of H5N8 clade 2.3.4.4 viruses and not related to the Asian H5N6 viruses that caused human infections.

In 2014 and 2016, outbreaks of highly pathogenic avian influenza (HPAI) subtype H5N8 clade 2.3.4.4 were observed among wild birds and domestic poultry worldwide (*1*) and in the Netherlands (*2**–**4*). Transcontinental spread of these viruses, and that of the earlier HPAI H5N1 virus (goose/Guangdong lineage) (*5*), has been linked to dissemination by migratory wild birds (*6*). A novel group B HPAI H5N6 virus (*7*) was detected in wild birds and commercial poultry in the Netherlands in December 2017. On December 6–7, 2017, meat ducks on a 16,400-duck farm in the municipality of Biddinghuizen, the Netherlands, began dying at an exponentially increasing rate (Figure 1; Technical Appendix Figure 1). The duck farm consisted of 2 barns, each housing ≈8,200 ducks. One-day-old ducklings started production in barn 1 on November 9 and in barn 2 on December 7. Mean water intake of ducks in barn 1 dropped by 8.5% during December 4–5. Mean feed intake dropped by 4.3% during December 3–5. Recording ended on December 5. On December 7, the clinical signs observed in barn 1, in addition to sudden death, were watery diarrhea, conjunctivitis, and nervous disorders. The following clinical signs were checked, but absent: edema (of the neck, head, and eyes); cyanosis (in the comb, wattle, and feet); hemorrhagic conjunctivae; and respiratory problems. No clinical signs were observed in the ducklings in barn 2.

**Figure 1:**
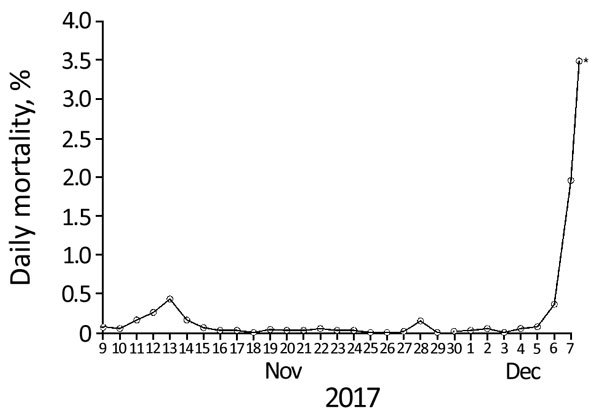
Daily mortality rate (% of ducks) in ≈8,000 ducks in barn 1 of the case flock during outbreak of highly pathogenic avian influenza A(H5N6) in the Netherlands, 2017. The farmer counted the dead ducks every morning. During clinical inspection on the last day, an additional number of dead ducks was counted (12 h after the farmer’s morning count); the asterisk (*) indicates the total number of dead ducks at the end of the day on December 7 (farmer count + clinical inspection count).

## The Study

We collected swab samples from the trachea and cloaca of clinically affected ducks for diagnostic testing. The samples tested positive by real-time PCR on the matrix gene (*3*) and H5-PCR (*8*), demonstrating notifiable avian influenza A subtype H5 virus. We performed hemagglutinin (HA) and neuraminidase (NA) sequence analysis (*3*), which showed a HA cleavage site with polybasic properties PLREKRRKR*GLF, and subtyped the virus as HPAI subtype H5N6 on December 8. The intravenous pathogenicity index determined in 6-week-old chickens for the novel H5N6 virus was 2.99, similar to that of the 2016 H5N8 subtype, confirming the high pathogenicity of the H5N6 subtype. 

The farm was located in a water-rich area, densely populated with wild waterbirds. Several mute swans (*Cygnus olor*) and a tufted duck (*Aythya fuligula*) were found dead in this area (Technical Appendix Figure 1), and tested positive for HPAI H5N6 on December 11.

Since 2013, HPAI H5N6 viruses have emerged in poultry and caused sporadic infections in humans in Asia, raising global concerns regarding their potential as human pandemic threats. H5N6 viruses constitute >34 distinct genotypes, of which 4 were detected in humans (*9*). To genetically characterize the novel H5N6 subtype influenza virus detected in the Netherlands, we sequenced the full genome of the viruses found at the duck farm, and in the 2 mute swans and the tufted duck (GISAID [http://platform.gisaid.org] accession nos. EPI ISL 287907, EPI ISL 288409, EPI ISL 288410, and EPI ISL 288412), as described previously (*4*). Database searches (GISAID and GenBank) showed that these viruses are highly similar to the HPAI H5N8 clade 2.3.4.4 viruses, which were detected previously in wild birds at the Russia–Mongolia border in May 2016 (*10*), for the gene segments polymerase basic 1 (PB1), polymerase acidic (PA), HA, nucleoprotein (NP), matrix protein (MP), and nonstructural protein (NS) (Table). The polymerase basic 2 (PB2) and NA segments shared sequence similarity with Eurasian low pathogenicity avian influenza (LPAI) viruses. Moreover, the N6 gene of the H5N6 viruses found in the Netherlands showed high homology to those detected in Greece in February and in Japan and Taiwan in November–December 2017.

**Table:** Genetic composition of the HPAI H5N6 virus isolated in the Netherlands, 2017*

Virus segment	GISAID no.†	Identity, %	Origin
Polymerase basic 2			
A/mallard duck/Netherlands/15/2011 (H6N8)	EPI889820	97	European LPAI
A/Eurasian teal/Netherlands/1/2011 (H3N8)	EPI889410	97	
A/mallard duck/Netherlands/20/2011 (H6N8)	EPI889594	97	
A/greater white-fronted goose/Netherlands/6/2011 (H6N8)	EPI1010712	97	
Polymerase basic 1			
A/chicken/Kalmykia/2643/2016 (H5N8)	EPI909458	99	H5N8 HPAI 2016
A/gadwall/Kurgan/2442/2016 (H5N8)	EPI961447	99	
A/T_Dk/NL-Werkendam/16014159–001/2016 (H5N5)	EPI1117251	98	
A/T_Dk/NL-Zeewolde/16013976–005/2016 (A/H5N8	EPI1019844	98	
Polymerase			
A/T_Dk/NL-Monnickendam/16013865–006–008/2016 (H5N8)	EPI1019770	99	H5N8 HPAI 2016
A/magpie/NL-Volendam/16014331–002/2016 (H5N8)	EPI1019722	99	
A/L-bl-ba-gull/NL-Sovon/16014324–014/2016 (H5N8)	EPI1019706	99	
A/G_c_grebe/NL-Monnickendam/16013865–009–010/2016 (H5N8)	EPI1019650	99	
Hemagglutinin			
A/Eur_Wig/NL-Zoeterwoude/16015702–010/2016 (H5N8)	EPI1019638	99	H5N8 HPAI 2016
A/Eur_Wig/NL-Reeuwijk/16015903–003/2016 (H5N8)	EPI1019590	99	
A/Eur_Wig/NL-Leidschendam/16015697–007/2016 (H5N8)	EPI1019582	99	
A/Eur_Wig/NL-Gouda/16015824–001/2016 (H5N8)	EPI1019550	99	
Nucleoprotein			
A/gadwall/Kurgan/2442/2016 (H5N8)	EPI961450	99	H5N8 HPAI 2016
A/wild duck/Tatarstan/3059/2016 (H5N8)	EPI909453	99	
A/T_Dk/NL-Werkendam/16014159–001/2016 (H5N5)	EPI1117254	99	
A/mute swan/Kaliningrad/132/2017 (H5N8)	EPI1044548	99	
Neuraminidase			
A/chicken/Greece/39_2017b/2017 (H5N6)	EPI1122895	98	H5N6 HPAI 2017
A/spoonbill/Taiwan/DB645/2017 (H5N6)	EPI1119073	97	
A/barnacle goose/Netherlands/2/2014 (H3N6)	EPI1011098	97	European LPAI
A/mute swan/Shimane/3211A001/2017 (H5N6)	LC335983	97	
Matrix protein			
A/mulard_duck/Hungary/59163/2016 (H5N8)	EPI1032553	99	H5N8 HPAI 2016
A/mulard_duck/Hungary/62902/2016 (H5N8)	EPI1032527	99	
A/mulard_duck/Hungary/60369/2016 (H5N8)	EPI1032519	99	
A/goose/Hungary/59763/2016 (H5N8)	EPI1032511	99	
Nonstructural protein			
A/duck/Hungary/60441/2016 (H5N8)	EPI866979	99	H5N8 HPAI 2016
A/goose/Italy/17VIR6358–3/2017 (H5N8)	EPI1081973	99	
A/swan/Italy/17VIR7064–1/2017 (H5N8)	EPI1081921	99	
A/mulard duck/Hungary/59163/2016 (H5N8)	EPI1032556	99	

*HPAI, highly pathogenic avian influenza; LPAI, low pathogenicity avian influenza. †From GISAID EpiFlu database (http://platform.gisaid.org).

**Figure 2:**
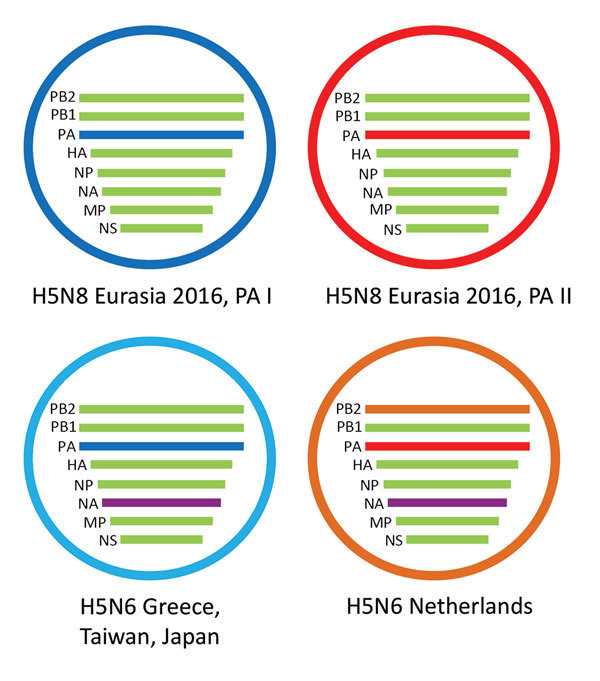
Schematic representation of the HPAI H5N6 reassortant virus detected in the Netherlands. Two variants of HPAI H5N8 were detected in 2016; they have different PA gene segments, called PA I and PA II. The novel virus evolved from H5N8 viruses having a PA II gene segment, but obtained both novel NA and PB2 gene segments. The H5N6 viruses detected in Greece, Japan, and Taiwan have evolved from H5N8 viruses that have a PA I gene segment and have an N6 segment similar to the virus detected in the Netherlands. HPAI, highly pathogenic avian influenza; PB, polymerase basic; PA, polymerase acidic; HA, hemagglutinin; NP, nucleoprotein; NA, neuraminidase; MP, matrix protein; NS, nonstructural protein.

To study the origin of the H5N6 virus detected in the Netherlands in December 2017, we performed a detailed phylogenetic analysis for all gene segments individually (Technical Appendix Figure 2). This analysis shows that the novel H5N6 virus is genetically distinct from human H5N6 viruses found in China. The PB1, PA, HA, NP, MP, and NS gene segments are closely related to HPAI H5N8 viruses detected in Europe in 2016 (Technical Appendix Figure 2, panels B–E, G, H). In contrast, the PB2 and NA genes are most closely related to Eurasian LPAI viruses (Technical Appendix Figure 2, panels A, F). Of note, the N6 segment of the virus in the Netherlands is closely related to, but distinct from, that of the H5N6 viruses detected in Greece, Japan, and Taiwan in 2017. Furthermore, the virus in the Netherlands has PB2 and PA segments that are distinct from those found in the viruses from Greece, Japan, and Taiwan (Figure 2). These results indicate that H5N6 virus in the Netherlands is a reassortant of the HPAI H5N8 subtype that obtained novel PB2 and NA segments.

To explain the emergence of the novel H5N6 virus, we performed molecular dating using the Bayesian skyline coalescent model in BEAST version 1.8 software (http://beast.community/beast; Technical Appendix Figure 3) and calculated the time to most recent common ancestor for the HA and NA gene segments (Technical Appendix Table 1). For the H5 segment, the viruses in the Netherlands, Greece, Taiwan, and Japan share a common ancestor with HPAI H5N8, which was dated in January–September 2016 (Technical Appendix Figure 3, panel A [node 1]). For the N6 segment, the common ancestor of the viruses in the Netherlands, Greece, Taiwan, and Japan was dated in December 2014–July 2016 (Technical Appendix Figure 3, panel B [node 2]). The novel H5N6 virus probably arose by reassortment of HPAI H5N8 and descendants of LPAI A/barnacle goose/Netherlands/2014 (node 1), sometime in 2015–2016. These results suggest that the reassortment event that generated the novel HPAI H5N6 virus probably did not occur within the Netherlands in 2017.

Finally, we analyzed the genome of the novel H5N6 virus for potential zoonotic signatures associated with increased human risk (Technical Appendix Table 2). We found that the virus has a typical avian receptor specificity and identified no sequence signatures associated with increased airborne transmission. In the MP and NS genes, we identified mutations that were associated with increased virulence, but similar mutations have been found in other H5 clade 2.3.4.4 viruses. Our analysis demonstrated that the virus may have reduced sensitivity to treatment with the antiviral drug oseltamivir.

## Conclusions

A novel reassortant HPAI H5N6 virus affected wild birds and commercial poultry in the Netherlands in December 2017. Phylogenetic analysis demonstrated that the virus is related to the HPAI H5N8 clade 2.3.4.4 viruses but contains novel PB2 and NA segments derived from Eurasian LPAI viruses. The N6 gene segment is related to that of HPAI H5N6 viruses found in Greece, Japan, and Taiwan, for which a common ancestor was estimated around November 2015. In addition, the H5N6 virus in the Netherlands differs from that in Greece by the PA and PB2 gene segments. This suggests that the H5N6 virus in the Netherlands did not result from continued circulation of the virus in Greece (or Europe) that was detected in February 2017 but likely represents a separate introduction related to wild bird migration in fall 2017. The reassortment events may have occurred on breeding grounds in Siberia, where large numbers of wild birds congregate, and the virus may have spread by long-distance flights of infected migratory birds (*6*).

Phylogenetic analysis demonstrated that the virus is not related to the zoonotic Asian H5N6 strains that cause infections in humans. Furthermore, genetic analysis identified no sequence features related to increased human risk. There are no indications that mammals (such as humans) can be infected by the novel reassortant HPAI H5N6 viruses detected in the Netherlands, Greece, Japan, and Taiwan. We recommend further studies in mammals (ferrets or mice) to provide experimental data on the virulence for mammals.

Technical Appendix Additional information about the emergence of HPAI H5N6 virus in the Netherlands in December 2017.
