# Supplementary material for: Novel Highly Pathogenic Avian Influenza A(H5N6) Virus in the Netherlands, December 2017
Source: Emerg Infect Dis. 2018 Apr;24(4):770–3. doi: 10.3201/eid2404.172124 (PMC5875252; doi:10.3201/eid2404.172124)
Supplement: Technical Appendix — Additional information about the emergence of HPAI H5N6 virus in the Netherlands in December 2017. [file 17-2124-Techapp-s1.pdf]

Article DOI: https://doi.org/10.3201/eid2404.172124

Novel Highly Pathogenic Avian Influenza A(H5N6) Virus in the Netherlands, December 2017

Nancy Beerens, Guus Koch, Rene Heutink, Frank Harders, D.P. Edwin Vries, Cynthia Ho, Alex Bossers, Armin Elbers

Technical Appendix

Technical Appendix Table 1. Calculated time to the most recent common ancestor for selected segments of avian influenza (H5N6) virus with 95% credible interval and posterior value*

			95% HPD		Posterior
Segment	Node†	tMRCA	Earliest date	Latest date	value
NA	1	2013 Sep 07	2012 Jun 04	2014 Oct 01	0.9965
	2	2015 Nov 17	2014 Dec 10	2016 Jul 14	0.9989
	3	2016 May 24	2015 Aug 16	2016 Nov 23	0.9868
	4	2017 Oct 16	2017 Jul 07	2017 Dec 06	0.9988
HA	1	2016 Jun 01	2016 Jan 15	2016 Sep 24	0.9994
	2	2016 Jun 22	2016 Feb 20	2016 Oct 07	0.2298
	3	2016 Oct 08	2016 Jul 19	2016 Nov 22	0.9997
	4	2017 Jul 26	2017 Feb 26	2017 Oct 31	0.9950
	5	2017 Sep 14	2017 Jun 04	2017 Nov 20	0.9994

*HPD, highest posterior density interval; tMRCA, time to most recent common ancestor (median).
†Nodes of the time-scaled phylogenetic tree.

Technical Appendix Table 2. Analysis of sequence features in influenza viruses associated with increased human risk*

Protein	Position	Present	Increased
PB2	D256G	No	Replication
	Q591K	No	Replication
	E627K	No	Virulence
	D701N	No	Replication
	S714R	No	Replication
PB1†	V3A	No	Virulence
	H99Y	No	Airborne transmission
	K207R	No	Virulence
	N328K	No	Virulence
	I368V	No	Airborne transmission
	N375S	No	Virulence
PA	T85I	No	Replication
	T97I	No	Virulence
	G186M	No	Replication
	L336S	No	Replication
	S409N	No	Human transmission
	K615N	No	Replication
	G631S	No	Virulence
H5	Various residues	No	Binding to α2–6
	119Y, 172A, 238L, 240S	No	Airborne transmission
	450K, 459K, 403I	No	Acid stability
NP	R99K	No	Airborne transmission
	N319K	No	Replication
	S345N	No	Airborne transmission
	Q357K	No	Virulence
N6	V116A	No	Resistance to oseltamivir, zanamivir
	E119V	No	Resistance to oseltamivir, zanamivir
	Q136K	No	Resistance to zanamivir
	D199N	Yes	Resistance to oseltamivir

Protein	Position	Present	Increased
	H275Y	No	Resistance to oseltamivir, zanamivir
M1	N30D	Yes‡	Virulence
	T139A	No	Virulence
	Y215A	Yes‡	Virulence
NS1	P42S	Yes‡	Virulence
	D92E	No	Virulence
	L103F	Yes‡	Virulence
	I106M	Yes‡	Virulence
	80–84	No	Virulence

*PB, polymerase basic; PA, polymerase; HA, hemagglutinin; NA, neuraminidase; M, matrix; NP, nucleoprotein, NS, nonstructural protein.
†PB1-F2 truncation amino acids 12–90
‡Sequence features of H5 Clade 2.3.4.4 viruses

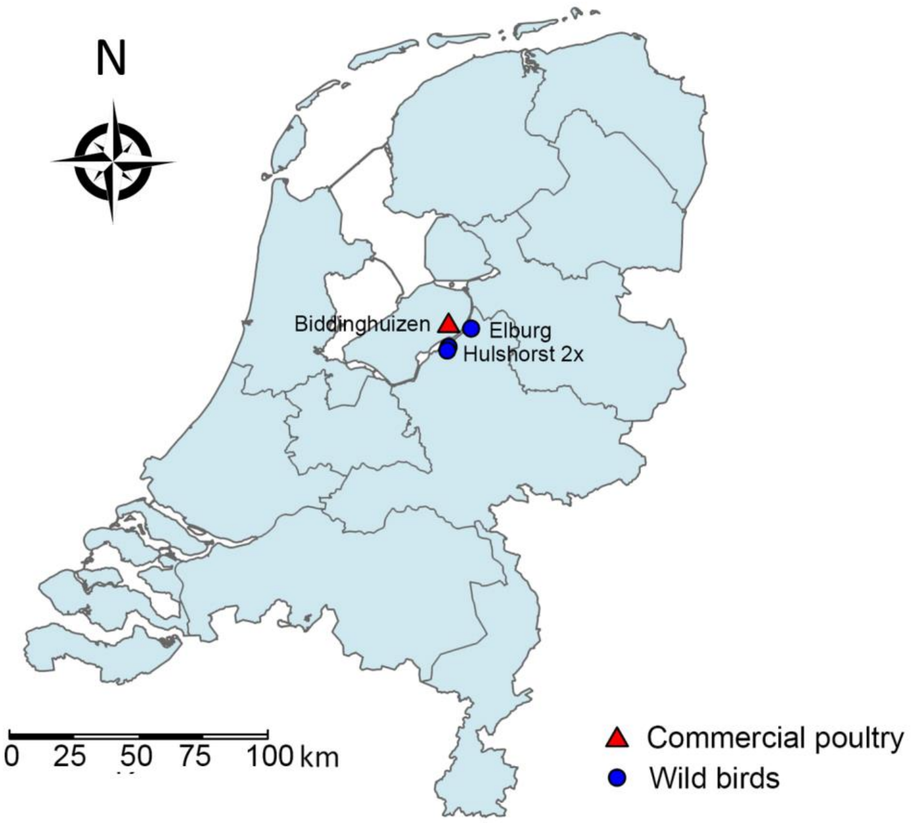

Technical Appendix Figure 1. Map of the Netherlands, showing locations of the duck farm (red) and the dead wild birds infected with HPAI H5N6 (blue).

A

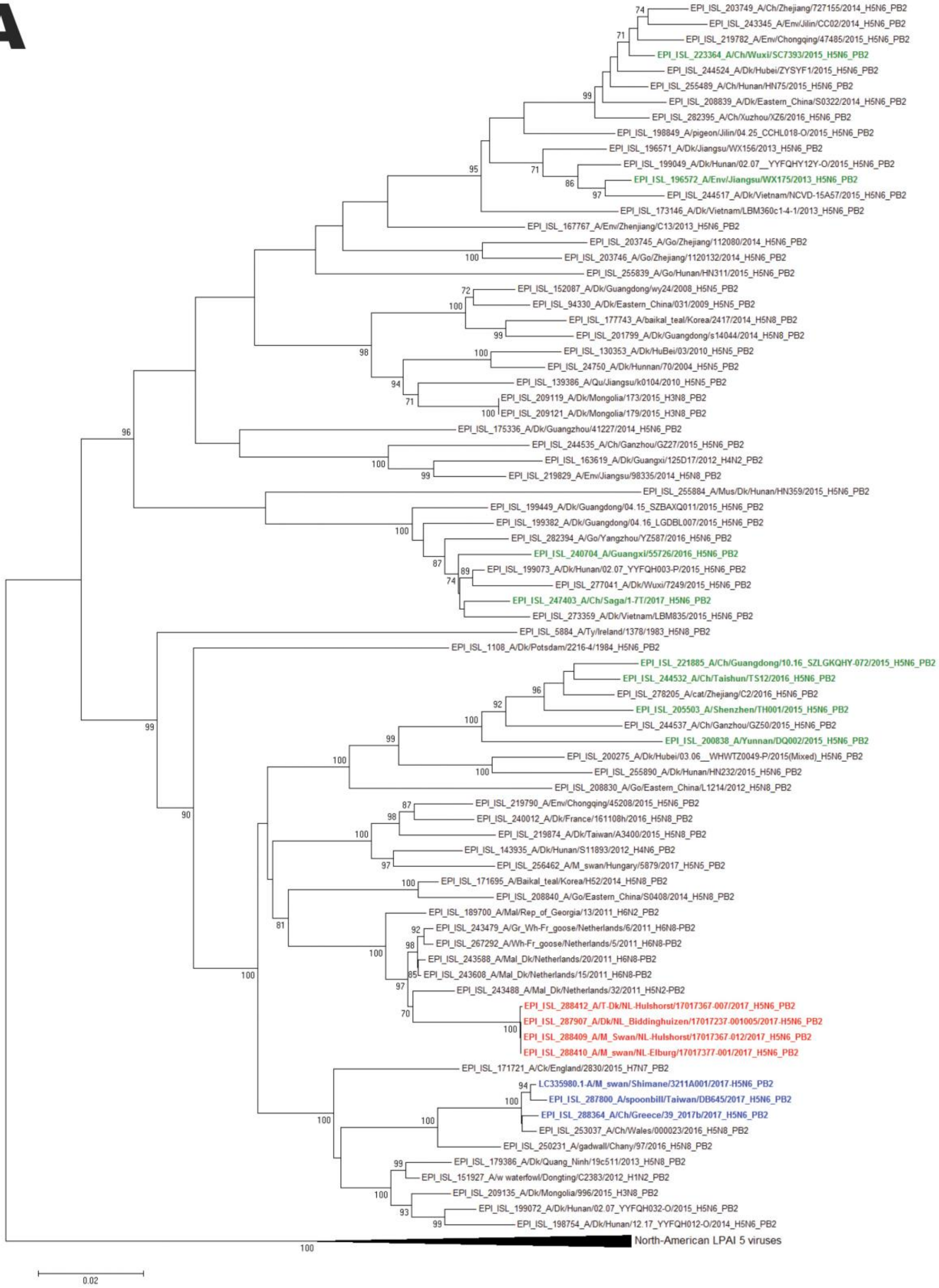

EPI_ISL_203749_A/Ch/Zhejiang/727155/2014_H5N6_PB2
EPI_ISL_243345_A/Env/Jilin/CC02/2014_H5N6_PB2
EPI_ISL_219782_A/Env/Chongqing/47485/2015_H5N6_PB2
EPI_ISL_223364_A/Ch/Wuxi/SC7393/2015_H5N6_PB2
EPI_ISL_244524_A/Dk/Hubei/ZYSYF1/2015_H5N6_PB2
EPI_ISL_255489_A/Ch/Hunan/HN75/2015_H5N6_PB2
EPI_ISL_208839_A/Dk/Eastern_China/S0322/2014_H5N6_PB2
EPI_ISL_282395_A/Ch/Xuzhou/XZ6/2016_H5N6_PB2
EPI_ISL_198849_A/pigeon/Jilin/04.25_CCHL018-O/2015_H5N6_PB2
EPI_ISL_196571_A/Dk/Jiangsu/WX156/2013_H5N6_PB2
EPI_ISL_199049_A/Dk/Hunan/02.07__YYFQHY12Y-O/2015_H5N6_PB2
EPI_ISL_196572_A/Env/Jiangsu/WX175/2013_H5N6_PB2
EPI_ISL_244517_A/Dk/Vietnam/NCVD-15A57/2015_H5N6_PB2
EPI_ISL_173146_A/Dk/Vietnam/LBM360c1-4-1/2013_H5N6_PB2
EPI_ISL_167767_A/Env/Zhenjiang/C13/2013_H5N6_PB2
EPI_ISL_203745_A/Go/Zhejiang/112080/2014_H5N6_PB2
EPI_ISL_203746_A/Go/Zhejiang/1120132/2014_H5N6_PB2
EPI_ISL_255839_A/Go/Hunan/HN311/2015_H5N6_PB2
EPI_ISL_152087_A/Dk/Guangdong/wy24/2008_H5N5_PB2
EPI_ISL_94330_A/Dk/Eastern_China/031/2009_H5N5_PB2
EPI_ISL_177743_A/baikal_teal/Korea/2417/2014_H5N8_PB2
EPI_ISL_201799_A/Dk/Guangdong/s14044/2014_H5N8_PB2
EPI_ISL_130353_A/Dk/HuBei/03/2010_H5N5_PB2
EPI_ISL_24750_A/Dk/Hunnan/70/2004_H5N5_PB2
EPI_ISL_139386_A/Qu/Jiangsu/k0104/2010_H5N5_PB2
EPI_ISL_209119_A/Dk/Mongolia/173/2015_H3N8_PB2
EPI_ISL_209121_A/Dk/Mongolia/179/2015_H3N8_PB2
EPI_ISL_175336_A/Dk/Guangzhou/41227/2014_H5N6_PB2
EPI_ISL_244535_A/Ch/Ganzhou/GZ27/2015_H5N6_PB2
EPI_ISL_163619_A/Dk/Guangxi/125D17/2012_H4N2_PB2
EPI_ISL_219829_A/Env/Jiangsu/98335/2014_H5N8_PB2
EPI_ISL_255884_A/Mus/Dk/Hunan/HN359/2015_H5N6_PB2
EPI_ISL_199449_A/Dk/Guangdong/04.15_SZBAXQ011/2015_H5N6_PB2
EPI_ISL_199382_A/Dk/Guangdong/04.16_LGDBL007/2015_H5N6_PB2
EPI_ISL_282394_A/Go/Yangzhou/YZ587/2016_H5N6_PB2
EPI_ISL_240704_A/Guangxi/55726/2016_H5N6_PB2
EPI_ISL_199073_A/Dk/Hunan/02.07_YYFQH003-P/2015_H5N6_PB2
EPI_ISL_277041_A/Dk/Wuxi/7249/2015_H5N6_PB2
EPI_ISL_247403_A/Ch/Saga/1-7T/2017_H5N6_PB2
EPI_ISL_273359_A/Dk/Vietnam/LBM835/2015_H5N6_PB2
EPI_ISL_5884_A/Ty/Ireland/1378/1983_H5N8_PB2
EPI_ISL_1108_A/Dk/Potsdam/2216-4/1984_H5N6_PB2
EPI_ISL_221885_A/Ch/Guangdong/10.16_SZLGKQHY-072/2015_H5N6_PB2
EPI_ISL_244532_A/Ch/Taishun/TS12/2016_H5N6_PB2
EPI_ISL_278205_A/cat/Zhejiang/C2/2016_H5N6_PB2
EPI_ISL_205503_A/Shenzhen/TH001/2015_H5N6_PB2
EPI_ISL_244537_A/Ch/Ganzhou/GZ50/2015_H5N6_PB2
EPI_ISL_200838_A/Yunnan/DQ002/2015_H5N6_PB2
EPI_ISL_200275_A/Dk/Hubei/03.06__WHWTZ0049-P/2015(Mixed)_H5N6_PB2
EPI_ISL_255890_A/Dk/Hunan/HN232/2015_H5N6_PB2
EPI_ISL_208830_A/Go/Eastern_China/L1214/2012_H5N8_PB2
EPI_ISL_219790_A/Env/Chongqing/45208/2015_H5N6_PB2
EPI_ISL_240012_A/Dk/France/161108h/2016_H5N8_PB2
EPI_ISL_219874_A/Dk/Taiwan/A3400/2015_H5N8_PB2
EPI_ISL_143935_A/Dk/Hunan/S11893/2012_H4N6_PB2
EPI_ISL_256462_A/M_swan/Hungary/5879/2017_H5N5_PB2
EPI_ISL_171695_A/Baikal_teal/Korea/H52/2014_H5N8_PB2
EPI_ISL_208840_A/Go/Eastern_China/S0408/2014_H5N8_PB2
EPI_ISL_189700_A/Mal/Rep_of_Georgia/13/2011_H6N2_PB2
EPI_ISL_243479_A/Gr_Wh-Fr_goose/Netherlands/6/2011_H6N8-PB2
EPI_ISL_267292_A/Wh-Fr_goose/Netherlands/5/2011_H6N8-PB2
EPI_ISL_243588_A/Mal_Dk/Netherlands/20/2011_H6N8-PB2
EPI_ISL_243608_A/Mal_Dk/Netherlands/15/2011_H6N8-PB2
EPI_ISL_243488_A/Mal_Dk/Netherlands/32/2011_H5N2-PB2
EPI_ISL_288412_A/T-Dk/NL-Hulshorst/17017367-007/2017_H5N6_PB2
EPI_ISL_287907_A/Dk/NL_Biddinghuizen/17017237-001005/2017-H5N6_PB2
EPI_ISL_288409_A/M_Swan/NL-Hulshorst/17017367-012/2017_H5N6_PB2
EPI_ISL_288410_A/M_swan/NL-Elburg/17017377-001/2017_H5N6_PB2
EPI_ISL_171721_A/Ck/England/2830/2015_H7N7_PB2
LC335980.1-A/M_swan/Shimane/3211A001/2017-H5N6_PB2
EPI_ISL_287800_A/spoonbill/Taiwan/DB645/2017_H5N6_PB2
EPI_ISL_288364_A/Ch/Greece/39_2017b/2017_H5N6_PB2
EPI_ISL_253037_A/Ch/Wales/000023/2016_H5N8_PB2
EPI_ISL_250231_A/gadwall/Chany/97/2016_H5N8_PB2
EPI_ISL_179386_A/Dk/Quang_Ninh/19c511/2013_H5N8_PB2
EPI_ISL_151927_A/w waterfowl/Dongting/C2383/2012_H1N2_PB2
EPI_ISL_209135_A/Dk/Mongolia/996/2015_H3N8_PB2
EPI_ISL_199072_A/Dk/Hunan/02.07_YYFQH032-O/2015_H5N6_PB2
EPI_ISL_198754_A/Dk/Hunan/12.17_YYFQH012-O/2014_H5N6_PB2
North-American LPAI 5 viruses
0.02

B

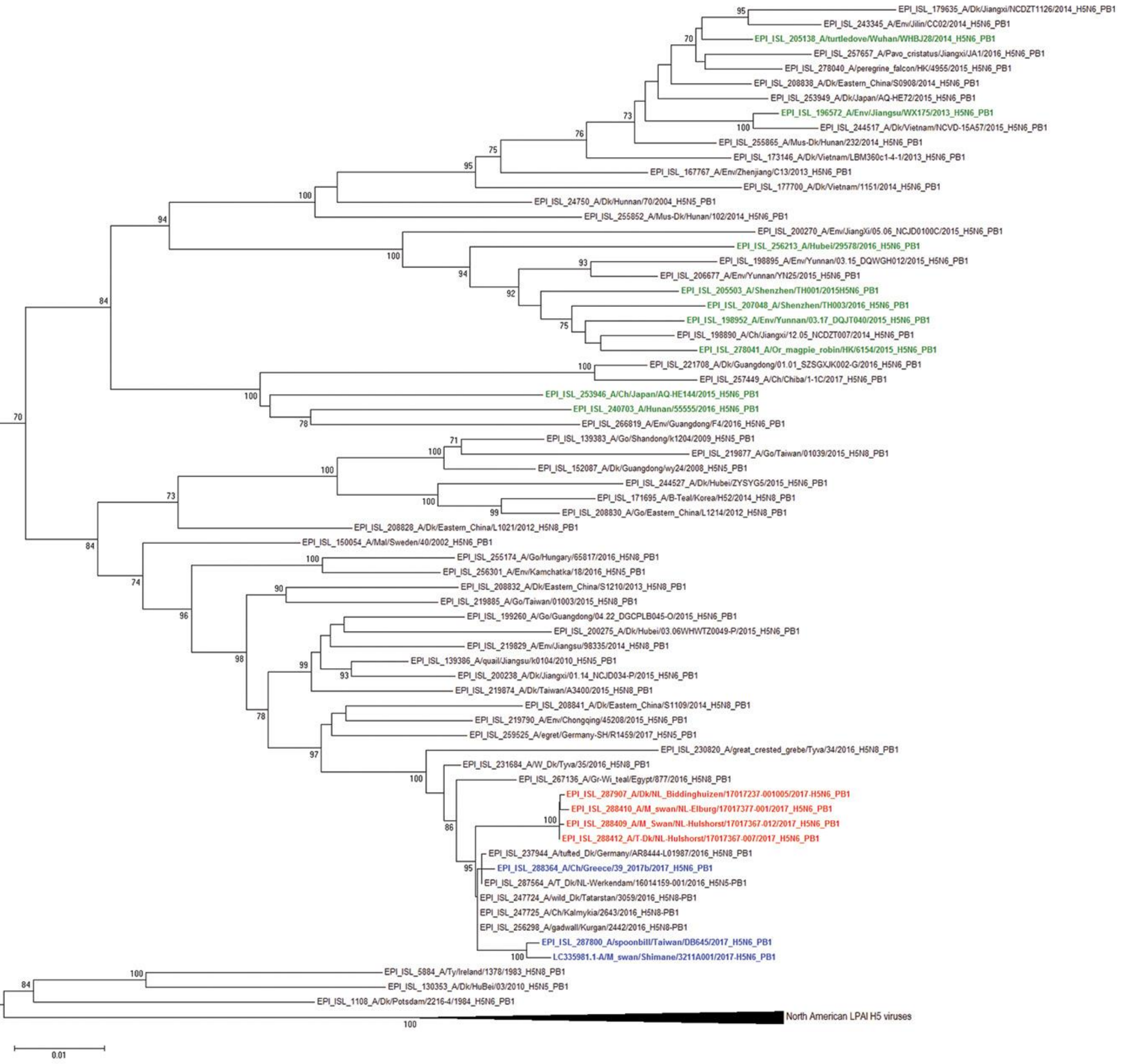
EPI_ISL_179635_A/Dk/Jiangxi/NCDZT1126/2014_H5N6_PB1
EPI_ISL_243345_A/Env/Jilin/CC02/2014_H5N6_PB1
EPI_ISL_205138_A/turtledove/Wuhan/WHBJ28/2014_H5N6_PB1
EPI_ISL_257657_A/Pavo_cristatus/Jiangxi/JA1/2016_H5N6_PB1
EPI_ISL_278040_A/peregrine_falcon/HK/4955/2015_H5N6_PB1
EPI_ISL_208838_A/Dk/Eastern_China/S0908/2014_H5N6_PB1
EPI_ISL_253949_A/Dk/Japan/AQ-HE72/2015_H5N6_PB1
EPI_ISL_196572_A/Env/Jiangsu/WX175/2013_H5N6_PB1
EPI_ISL_244517_A/Dk/Vietnam/NCVD-15A57/2015_H5N6_PB1
EPI_ISL_255865_A/Mus-Dk/Hunan/232/2014_H5N6_PB1
EPI_ISL_173146_A/Dk/Vietnam/LBM360c1-4-1/2013_H5N6_PB1
EPI_ISL_167767_A/Env/Zhenjiang/C13/2013_H5N6_PB1
EPI_ISL_177700_A/Dk/Vietnam/1151/2014_H5N6_PB1
EPI_ISL_24750_A/Dk/Hunnan/70/2004_H5N5_PB1
EPI_ISL_255852_A/Mus-Dk/Hunan/102/2014_H5N6_PB1
EPI_ISL_200270_A/Env/JiangXi/05.06_NCJD0100C/2015_H5N6_PB1
EPI_ISL_256213_A/Hubei/29578/2016_H5N6_PB1
EPI_ISL_198895_A/Env/Yunnan/03.15_DQWGH012/2015_H5N6_PB1
EPI_ISL_206677_A/Env/Yunnan/YN25/2015_H5N6_PB1
EPI_ISL_205503_A/Shenzhen/TH001/2015H5N6_PB1
EPI_ISL_207048_A/Shenzhen/TH003/2016_H5N6_PB1
EPI_ISL_198952_A/Env/Yunnan/03.17_DQJT040/2015_H5N6_PB1
EPI_ISL_198890_A/Ch/Jiangxi/12.05_NCDZT007/2014_H5N6_PB1
EPI_ISL_278041_A/Or_magpie_robin/HK/6154/2015_H5N6_PB1
EPI_ISL_221708_A/Dk/Guangdong/01.01_SZSGXJK002-G/2016_H5N6_PB1
EPI_ISL_257449_A/Ch/Chiba/1-1C/2017_H5N6_PB1
EPI_ISL_253946_A/Ch/Japan/AQ-HE144/2015_H5N6_PB1
EPI_ISL_240703_A/Hunan/55555/2016_H5N6_PB1
EPI_ISL_266819_A/Env/Guangdong/F4/2016_H5N6_PB1
EPI_ISL_139383_A/Go/Shandong/k1204/2009_H5N5_PB1
EPI_ISL_219877_A/Go/Taiwan/01039/2015_H5N8_PB1
EPI_ISL_152087_A/Dk/Guangdong/wy24/2008_H5N5_PB1
EPI_ISL_244527_A/Dk/Hubei/ZYSYG5/2015_H5N6_PB1
EPI_ISL_171695_A/B-Teal/Korea/H52/2014_H5N8_PB1
EPI_ISL_208830_A/Go/Eastern_China/L1214/2012_H5N8_PB1
EPI_ISL_208828_A/Dk/Eastern_China/L1021/2012_H5N8_PB1
EPI_ISL_150054_A/Mal/Sweden/40/2002_H5N6_PB1
EPI_ISL_255174_A/Go/Hungary/65817/2016_H5N8_PB1
EPI_ISL_256301_A/Env/Kamchatka/18/2016_H5N5_PB1
EPI_ISL_208832_A/Dk/Eastern_China/S1210/2013_H5N8_PB1
EPI_ISL_219885_A/Go/Taiwan/01003/2015_H5N8_PB1
EPI_ISL_199260_A/Go/Guangdong/04.22_DGCPLB045-O/2015_H5N6_PB1
EPI_ISL_200275_A/Dk/Hubei/03.06WHWTZ0049-P/2015_H5N6_PB1
EPI_ISL_219829_A/Env/Jiangsu/98335/2014_H5N8_PB1
EPI_ISL_139386_A/quail/Jiangsu/k0104/2010_H5N5_PB1
EPI_ISL_200238_A/Dk/Jiangxi/01.14_NCJD034-P/2015_H5N6_PB1
EPI_ISL_219874_A/Dk/Taiwan/A3400/2015_H5N8_PB1
EPI_ISL_208841_A/Dk/Eastern_China/S1109/2014_H5N8_PB1
EPI_ISL_219790_A/Env/Chongqing/45208/2015_H5N6_PB1
EPI_ISL_259525_A/egret/Germany-SH/R1459/2017_H5N5_PB1
EPI_ISL_230820_A/great_crested_grebe/Tyva/34/2016_H5N8_PB1
EPI_ISL_231684_A/W_Dk/Tyva/35/2016_H5N8_PB1
EPI_ISL_267136_A/Gr-Wi_teal/Egypt/877/2016_H5N8_PB1
EPI_ISL_287907_A/Dk/NL_Biddinghuizen/17017237-001005/2017-H5N6_PB1
EPI_ISL_288410_A/M_swan/NL-Elburg/17017377-001/2017_H5N6_PB1
EPI_ISL_288409_A/M_Swan/NL-Hulshorst/17017367-012/2017_H5N6_PB1
EPI_ISL_288412_A/T-Dk/NL-Hulshorst/17017367-007/2017_H5N6_PB1
EPI_ISL_237944_A/tufted_Dk/Germany/AR8444-L01987/2016_H5N8_PB1
EPI_ISL_288364_A/Ch/Greece/39_2017b/2017_H5N6_PB1
EPI_ISL_287564_A/T_Dk/NL-Werkendam/16014159-001/2016_H5N5-PB1
EPI_ISL_247724_A/wild_Dk/Tatarstan/3059/2016_H5N8-PB1
EPI_ISL_247725_A/Ch/Kalmykia/2643/2016_H5N8-PB1
EPI_ISL_256298_A/gadwall/Kurgan/2442/2016_H5N8-PB1
EPI_ISL_287800_A/spoonbill/Taiwan/DB645/2017_H5N6_PB1
LC335981.1-A/M_swan/Shimane/3211A001/2017-H5N6_PB1
EPI_ISL_5884_A/Ty/Ireland/1378/1983_H5N8_PB1
EPI_ISL_130353_A/Dk/HuBei/03/2010_H5N5_PB1
EPI_ISL_1108_A/Dk/Potsdam/2216-4/1984_H5N6_PB1
North American LPAI H5 viruses
0.01

c

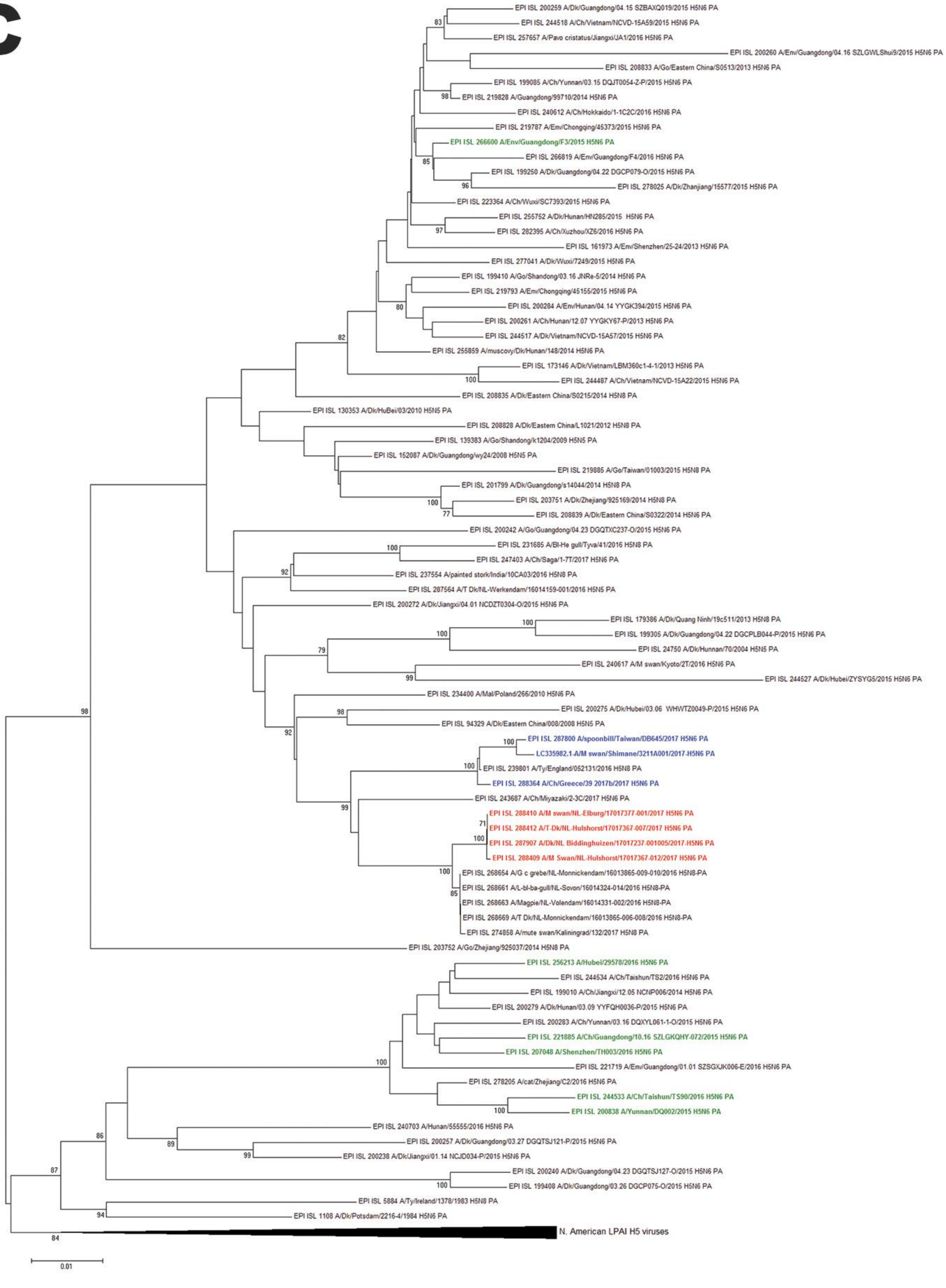
EPI ISL 200259 A/Dk/Guangdong/04.15 SZBAXQ019/2015 H5N6 PA
EPI ISL 244518 A/Ch/Vietnam/NCVD-15A59/2015 H5N6 PA
EPI ISL 257657 A/Pavo cristatus/Jiangxi/JA1/2016 H5N6 PA
EPI ISL 200260 A/Env/Guangdong/04.16 SZLGWLShui9/2015 H5N6 PA
EPI ISL 208833 A/Go/Eastern China/S0513/2013 H5N6 PA
EPI ISL 199085 A/Ch/Yunnan/03.15 DQJT0054-Z-P/2015 H5N6 PA
EPI ISL 219828 A/Guangdong/99710/2014 H5N6 PA
EPI ISL 240612 A/Ch/Hokkaido/1-1C2C/2016 H5N6 PA
EPI ISL 219787 A/Env/Chongqing/45373/2015 H5N6 PA
EPI ISL 266600 A/Env/Guangdong/F3/2015 H5N6 PA
EPI ISL 266819 A/Env/Guangdong/F4/2016 H5N6 PA
EPI ISL 199250 A/Dk/Guangdong/04.22 DGCP079-O/2015 H5N6 PA
EPI ISL 278025 A/Dk/Zhanjiang/15577/2015 H5N6 PA
EPI ISL 223364 A/Ch/Wuxi/SC7393/2015 H5N6 PA
EPI ISL 255752 A/Dk/Hunan/HN285/2015 H5N6 PA
EPI ISL 282395 A/Ch/Xuzhou/XZ6/2016 H5N6 PA
EPI ISL 161973 A/Env/Shenzhen/25-24/2013 H5N6 PA
EPI ISL 277041 A/Dk/Wuxi/7249/2015 H5N6 PA
EPI ISL 199410 A/Go/Shandong/03.16 JNRe-5/2014 H5N6 PA
EPI ISL 219793 A/Env/Chongqing/45155/2015 H5N6 PA
EPI ISL 200284 A/Env/Hunan/04.14 YYGK394/2015 H5N6 PA
EPI ISL 200261 A/Ch/Hunan/12.07 YYGKY67-P/2013 H5N6 PA
EPI ISL 244517 A/Dk/Vietnam/NCVD-15A57/2015 H5N6 PA
EPI ISL 255859 A/muscovy/Dk/Hunan/148/2014 H5N6 PA
EPI ISL 173146 A/Dk/Vietnam/LBM360c1-4-1/2013 H5N6 PA
EPI ISL 244487 A/Ch/Vietnam/NCVD-15A22/2015 H5N6 PA
EPI ISL 208835 A/Dk/Eastern China/S0215/2014 H5N8 PA
EPI ISL 130353 A/Dk/HuBei/03/2010 H5N5 PA
EPI ISL 208828 A/Dk/Eastern China/L1021/2012 H5N8 PA
EPI ISL 139383 A/Go/Shandong/k1204/2009 H5N5 PA
EPI ISL 152087 A/Dk/Guangdong/wy24/2008 H5N5 PA
EPI ISL 219885 A/Go/Taiwan/01003/2015 H5N8 PA
EPI ISL 201799 A/Dk/Guangdong/s14044/2014 H5N8 PA
EPI ISL 203751 A/Dk/Zhejiang/925169/2014 H5N8 PA
EPI ISL 208839 A/Dk/Eastern China/S0322/2014 H5N6 PA
EPI ISL 200242 A/Go/Guangdong/04.23 DGQTXC237-O/2015 H5N6 PA
EPI ISL 231685 A/Bl-He gull/Tyva/41/2016 H5N8 PA
EPI ISL 247403 A/Ch/Saga/1-7T/2017 H5N6 PA
EPI ISL 237554 A/painted stork/India/10CA03/2016 H5N8 PA
EPI ISL 287564 A/T Dk/NL-Werkendam/16014159-001/2016 H5N5 PA
EPI ISL 200272 A/Dk/Jiangxi/04.01 NCDZT0304-O/2015 H5N6 PA
EPI ISL 179386 A/Dk/Quang Ninh/19c511/2013 H5N8 PA
EPI ISL 199305 A/Dk/Guangdong/04.22 DGCPLB044-P/2015 H5N6 PA
EPI ISL 24750 A/Dk/Hunnan/70/2004 H5N5 PA
EPI ISL 240617 A/M swan/Kyoto/2T/2016 H5N6 PA
EPI ISL 244527 A/Dk/Hubei/ZYSYG5/2015 H5N6 PA
EPI ISL 234400 A/Mal/Poland/266/2010 H5N6 PA
EPI ISL 200275 A/Dk/Hubei/03.06 WHWTZ0049-P/2015 H5N6 PA
EPI ISL 94329 A/Dk/Eastern China/008/2008 H5N5 PA
EPI ISL 287800 A/spoonbill/Taiwan/DB645/2017 H5N6 PA
LC335982.1-A/M swan/Shimane/3211A001/2017-H5N6 PA
EPI ISL 239801 A/Ty/England/052131/2016 H5N8 PA
EPI ISL 288364 A/Ch/Greece/39 2017b/2017 H5N6 PA
EPI ISL 243687 A/Ch/Miyazaki/2-3C/2017 H5N6 PA
EPI ISL 288410 A/M swan/NL-Elburg/17017377-001/2017 H5N6 PA
EPI ISL 288412 A/T-Dk/NL-Hulshorst/17017367-007/2017 H5N6 PA
EPI ISL 287907 A/Dk/NL Biddinghuizen/17017237-001005/2017-H5N6 PA
EPI ISL 288409 A/M Swan/NL-Hulshorst/17017367-012/2017 H5N6 PA
EPI ISL 268654 A/G c grebe/NL-Monnickendam/16013865-009-010/2016 H5N8-PA
EPI ISL 268661 A/L-bl-ba-gull/NL-Sovon/16014324-014/2016 H5N8-PA
EPI ISL 268663 A/Magpie/NL-Volendam/16014331-002/2016 H5N8-PA
EPI ISL 268669 A/T Dk/NL-Monnickendam/16013865-006-008/2016 H5N8-PA
EPI ISL 274858 A/mute swan/Kaliningrad/132/2017 H5N8 PA
EPI ISL 203752 A/Go/Zhejiang/925037/2014 H5N8 PA
EPI ISL 256213 A/Hubei/29578/2016 H5N6 PA
EPI ISL 244534 A/Ch/Taishun/TS2/2016 H5N6 PA
EPI ISL 199010 A/Ch/Jiangxi/12.05 NCNP006/2014 H5N6 PA
EPI ISL 200279 A/Dk/Hunan/03.09 YYFQH0036-P/2015 H5N6 PA
EPI ISL 200283 A/Ch/Yunnan/03.16 DQXYL061-1-O/2015 H5N6 PA
EPI ISL 221885 A/Ch/Guangdong/10.16 SZLGKQHY-072/2015 H5N6 PA
EPI ISL 207048 A/Shenzhen/TH003/2016 H5N6 PA
EPI ISL 221719 A/Env/Guangdong/01.01 SZSGXJK006-E/2016 H5N6 PA
EPI ISL 278205 A/cat/Zhejiang/C2/2016 H5N6 PA
EPI ISL 244533 A/Ch/Taishun/TS90/2016 H5N6 PA
EPI ISL 200838 A/Yunnan/DQ002/2015 H5N6 PA
EPI ISL 240703 A/Hunan/55555/2016 H5N6 PA
EPI ISL 200257 A/Dk/Guangdong/03.27 DGQTSJ121-P/2015 H5N6 PA
EPI ISL 200238 A/Dk/Jiangxi/01.14 NCJD034-P/2015 H5N6 PA
EPI ISL 200240 A/Dk/Guangdong/04.23 DGQTSJ127-O/2015 H5N6 PA
EPI ISL 199408 A/Dk/Guangdong/03.26 DGCP075-O/2015 H5N6 PA
EPI ISL 5884 A/Ty/Ireland/1378/1983 H5N8 PA
EPI ISL 1108 A/Dk/Potsdam/2216-4/1984 H5N6 PA
N. American LPAI H5 viruses
0.01

D

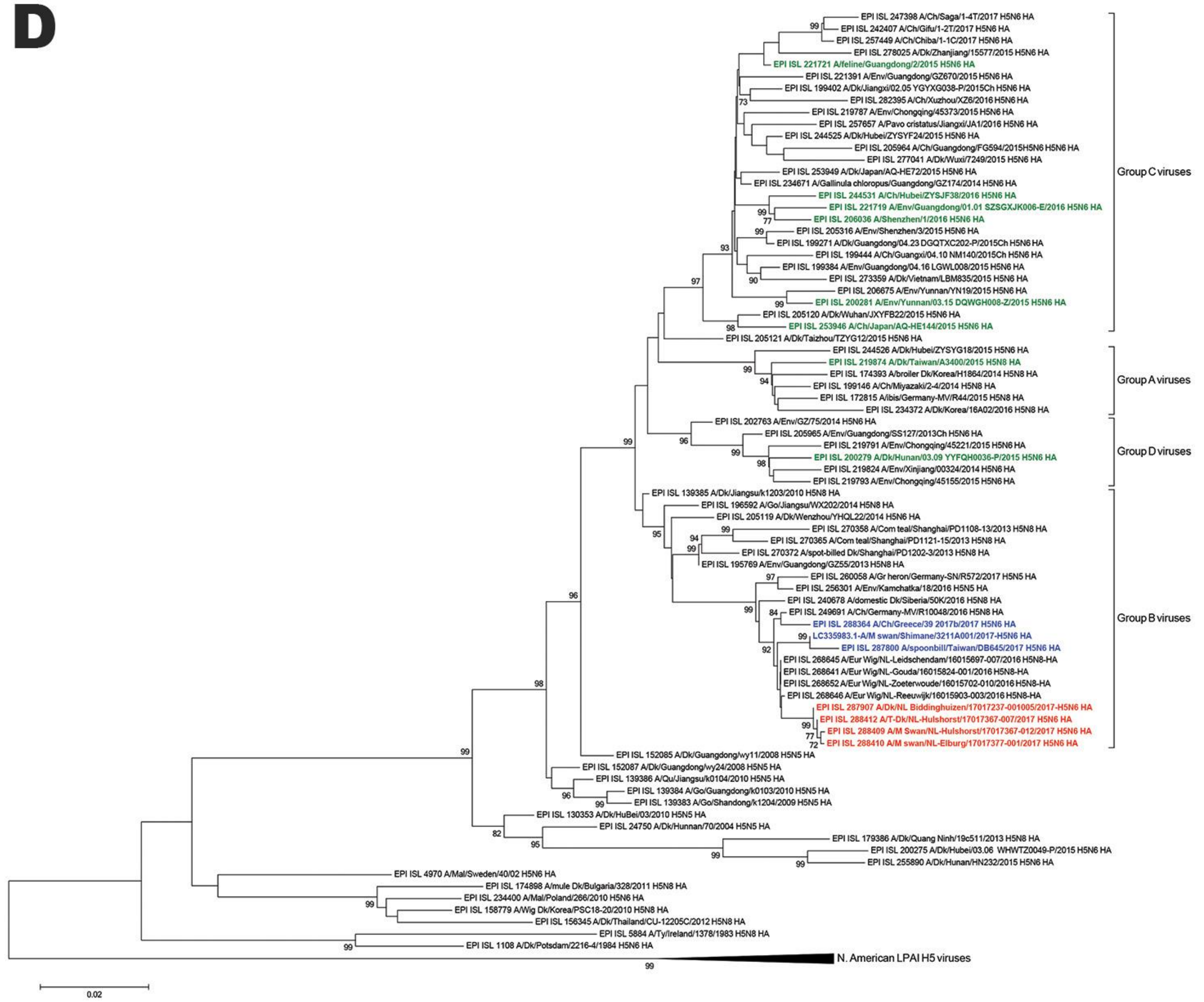
EPI ISL 247398 A/Ch/Saga/1-4T/2017 H5N6 HA
EPI ISL 242407 A/Ch/Gifu/1-2T/2017 H5N6 HA
EPI ISL 257449 A/Ch/Chiba/1-1C/2017 H5N6 HA
EPI ISL 278025 A/Dk/Zhanjiang/15577/2015 H5N6 HA
EPI ISL 221721 A/feline/Guangdong/2/2015 H5N6 HA
EPI ISL 221391 A/Env/Guangdong/GZ670/2015 H5N6 HA
EPI ISL 199402 A/Dk/Jiangxi/02.05 YGYXG038-P/2015Ch H5N6 HA
EPI ISL 282395 A/Ch/Xuzhou/XZ6/2016 H5N6 HA
EPI ISL 219787 A/Env/Chongqing/45373/2015 H5N6 HA
EPI ISL 257657 A/Pavo cristatus/Jiangxi/JA1/2016 H5N6 HA
EPI ISL 244525 A/Dk/Hubei/ZYSYF24/2015 H5N6 HA
EPI ISL 205964 A/Ch/Guangdong/FG594/2015H5N6 H5N6 HA
EPI ISL 277041 A/Dk/Wuxi/7249/2015 H5N6 HA
EPI ISL 253949 A/Dk/Japan/AQ-HE72/2015 H5N6 HA
EPI ISL 234671 A/Gallinula chloropus/Guangdong/GZ174/2014 H5N6 HA
EPI ISL 244531 A/Ch/Hubei/ZYSJF38/2016 H5N6 HA
EPI ISL 221719 A/Env/Guangdong/01.01 SZSGXJK006-E/2016 H5N6 HA
EPI ISL 206036 A/Shenzhen/1/2016 H5N6 HA
EPI ISL 205316 A/Env/Shenzhen/3/2015 H5N6 HA
EPI ISL 199271 A/Dk/Guangdong/04.23 DGQTXC202-P/2015Ch H5N6 HA
EPI ISL 199444 A/Ch/Guangxi/04.10 NM140/2015Ch H5N6 HA
EPI ISL 199384 A/Env/Guangdong/04.16 LGWL008/2015 H5N6 HA
EPI ISL 273359 A/Dk/Vietnam/LBM835/2015 H5N6 HA
EPI ISL 206675 A/Env/Yunnan/YN19/2015 H5N6 HA
EPI ISL 200281 A/Env/Yunnan/03.15 DQWGH008-Z/2015 H5N6 HA
EPI ISL 205120 A/Dk/Wuhan/JXYFB22/2015 H5N6 HA
EPI ISL 253946 A/Ch/Japan/AQ-HE144/2015 H5N6 HA
Group C viruses
EPI ISL 205121 A/Dk/Taizhou/TZYG12/2015 H5N6 HA
EPI ISL 244526 A/Dk/Hubei/ZYSYG18/2015 H5N6 HA
EPI ISL 219874 A/Dk/Taiwan/A3400/2015 H5N8 HA
EPI ISL 174393 A/broiler Dk/Korea/H1864/2014 H5N8 HA
EPI ISL 199146 A/Ch/Miyazaki/2-4/2014 H5N8 HA
EPI ISL 172815 A/ibis/Germany-MV/R44/2015 H5N8 HA
EPI ISL 234372 A/Dk/Korea/16A02/2016 H5N8 HA
Group A viruses
EPI ISL 202763 A/Env/GZ/75/2014 H5N6 HA
EPI ISL 205965 A/Env/Guangdong/SS127/2013Ch H5N6 HA
EPI ISL 219791 A/Env/Chongqing/45221/2015 H5N6 HA
EPI ISL 200279 A/Dk/Hunan/03.09 YYFQH0036-P/2015 H5N6 HA
EPI ISL 219824 A/Env/Xinjiang/00324/2014 H5N6 HA
EPI ISL 219793 A/Env/Chongqing/45155/2015 H5N6 HA
Group D viruses
EPI ISL 139385 A/Dk/Jiangsu/k1203/2010 H5N8 HA
EPI ISL 196592 A/Go/Jiangsu/WX202/2014 H5N8 HA
EPI ISL 205119 A/Dk/Wenzhou/YHQL22/2014 H5N6 HA
EPI ISL 270358 A/Com teal/Shanghai/PD1108-13/2013 H5N8 HA
EPI ISL 270365 A/Com teal/Shanghai/PD1121-15/2013 H5N8 HA
EPI ISL 270372 A/spot-billed Dk/Shanghai/PD1202-3/2013 H5N8 HA
EPI ISL 195769 A/Env/Guangdong/GZ55/2013 H5N8 HA
EPI ISL 260058 A/Gr heron/Germany-SN/R572/2017 H5N5 HA
EPI ISL 256301 A/Env/Kamchatka/18/2016 H5N5 HA
EPI ISL 240678 A/domestic Dk/Siberia/50K/2016 H5N8 HA
EPI ISL 249691 A/Ch/Germany-MV/R10048/2016 H5N8 HA
EPI ISL 288364 A/Ch/Greece/39 2017b/2017 H5N6 HA
LC335983.1-A/M swan/Shimane/3211A001/2017-H5N6 HA
EPI ISL 287800 A/spoonbill/Taiwan/DB645/2017 H5N6 HA
EPI ISL 268645 A/Eur Wig/NL-Leidschendam/16015697-007/2016 H5N8-HA
EPI ISL 268641 A/Eur Wig/NL-Gouda/16015824-001/2016 H5N8-HA
EPI ISL 268652 A/Eur Wig/NL-Zoeterwoude/16015702-010/2016 H5N8-HA
EPI ISL 268646 A/Eur Wig/NL-Reeuwijk/16015903-003/2016 H5N8-HA
EPI ISL 287907 A/Dk/NL Biddinghuizen/17017237-001005/2017-H5N6 HA
EPI ISL 288412 A/T-Dk/NL-Hulshorst/17017367-007/2017 H5N6 HA
EPI ISL 288409 A/M Swan/NL-Hulshorst/17017367-012/2017 H5N6 HA
EPI ISL 288410 A/M swan/NL-Elburg/17017377-001/2017 H5N6 HA
Group B viruses
EPI ISL 152085 A/Dk/Guangdong/wy11/2008 H5N5 HA
EPI ISL 152087 A/Dk/Guangdong/wy24/2008 H5N5 HA
EPI ISL 139386 A/Qu/Jiangsu/k0104/2010 H5N5 HA
EPI ISL 139384 A/Go/Guangdong/k0103/2010 H5N5 HA
EPI ISL 139383 A/Go/Shandong/k1204/2009 H5N5 HA
EPI ISL 130353 A/Dk/HuBei/03/2010 H5N5 HA
EPI ISL 24750 A/Dk/Hunnan/70/2004 H5N5 HA
EPI ISL 179386 A/Dk/Quang Ninh/19c511/2013 H5N8 HA
EPI ISL 200275 A/Dk/Hubei/03.06 WHWTZ0049-P/2015 H5N6 HA
EPI ISL 255890 A/Dk/Hunan/HN232/2015 H5N6 HA
EPI ISL 4970 A/Mal/Sweden/40/02 H5N6 HA
EPI ISL 174898 A/mule Dk/Bulgaria/328/2011 H5N8 HA
EPI ISL 234400 A/Mal/Poland/266/2010 H5N6 HA
EPI ISL 158779 A/Wig Dk/Korea/PSC18-20/2010 H5N8 HA
EPI ISL 156345 A/Dk/Thailand/CU-12205C/2012 H5N8 HA
EPI ISL 5884 A/Ty/Ireland/1378/1983 H5N8 HA
EPI ISL 1108 A/Dk/Potsdam/2216-4/1984 H5N6 HA
N. American LPAI H5 viruses
0.02

E

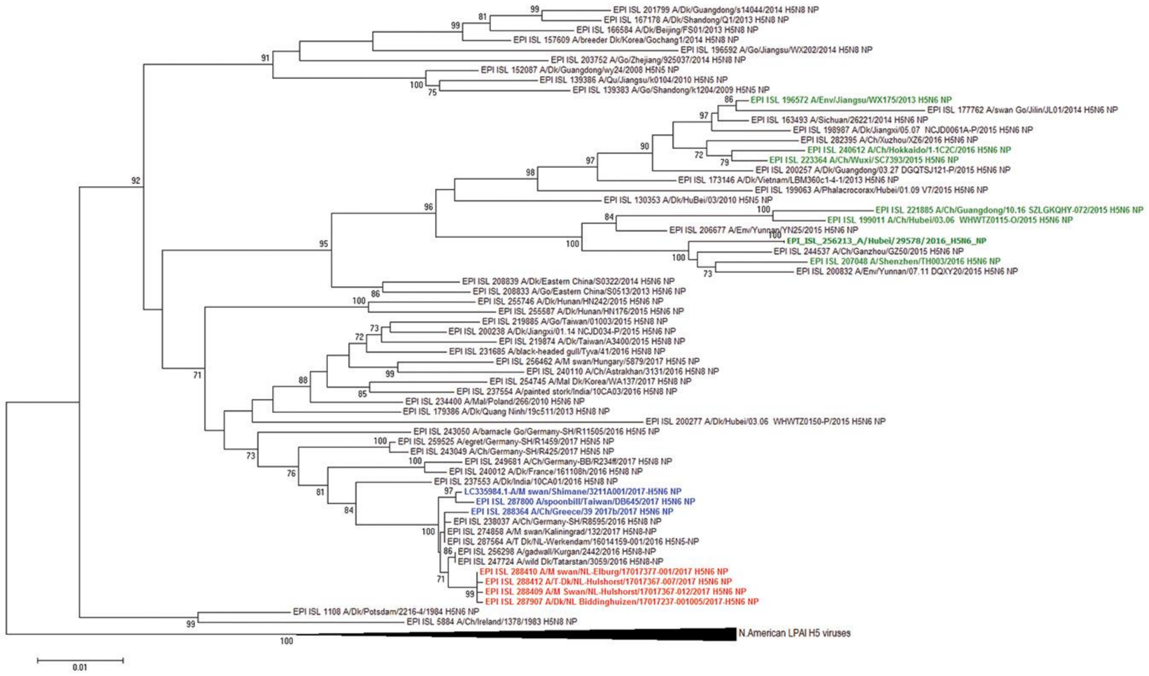
EPI ISL 201799 A/Dk/Guangdong/s14044/2014 H5N8 NP
EPI ISL 167178 A/Dk/Shandong/Q1/2013 H5N8 NP
EPI ISL 166584 A/Dk/Beijing/FS01/2013 H5N8 NP
EPI ISL 157609 A/breeder Dk/Korea/Gochang1/2014 H5N8 NP
EPI ISL 196592 A/Go/Jiangsu/WX202/2014 H5N8 NP
EPI ISL 203752 A/Go/Zhejiang/925037/2014 H5N8 NP
EPI ISL 152087 A/Dk/Guangdong/wy24/2008 H5N5 NP
EPI ISL 139386 A/Qu/Jiangsu/k0104/2010 H5N5 NP
EPI ISL 139383 A/Go/Shandong/k1204/2009 H5N5 NP
EPI ISL 196572 A/Env/Jiangsu/WX175/2013 H5N6 NP
EPI ISL 177762 A/swan Go/Jilin/JL01/2014 H5N6 NP
EPI ISL 163493 A/Sichuan/26221/2014 H5N6 NP
EPI ISL 198987 A/Dk/Jiangxi/05.07 NCJD0061A-P/2015 H5N6 NP
EPI ISL 282395 A/Ch/Xuzhou/XZ6/2016 H5N6 NP
EPI ISL 240612 A/Ch/Hokkaido/1-1C2C/2016 H5N6 NP
EPI ISL 223364 A/Ch/Wuxi/SC7393/2015 H5N6 NP
EPI ISL 200257 A/Dk/Guangdong/03.27 DGQTSJ121-P/2015 H5N6 NP
EPI ISL 173146 A/Dk/Vietnam/LBM360c1-4-1/2013 H5N6 NP
EPI ISL 199063 A/Phalacrocorax/Hubei/01.09 V7/2015 H5N6 NP
EPI ISL 130353 A/Dk/HuBei/03/2010 H5N5 NP
EPI ISL 221885 A/Ch/Guangdong/10.16 SZLGKQHY-072/2015 H5N6 NP
EPI ISL 199011 A/Ch/Hubei/03.06 WHWTZ0115-O/2015 H5N6 NP
EPI ISL 206677 A/Env/Yunnan/YN25/2015 H5N6 NP
EPI_ISL_256213_A/Hubei/29578/2016_H5N6_NP
EPI ISL 244537 A/Ch/Ganzhou/GZ50/2015 H5N6 NP
EPI ISL 207048 A/Shenzhen/TH003/2016 H5N6 NP
EPI ISL 200832 A/Env/Yunnan/07.11 DQXY20/2015 H5N6 NP
EPI ISL 208839 A/Dk/Eastern China/S0322/2014 H5N6 NP
EPI ISL 208833 A/Go/Eastern China/S0513/2013 H5N6 NP
EPI ISL 255746 A/Dk/Hunan/HN242/2015 H5N6 NP
EPI ISL 255587 A/Dk/Hunan/HN176/2015 H5N6 NP
EPI ISL 219885 A/Go/Taiwan/01003/2015 H5N8 NP
EPI ISL 200238 A/Dk/Jiangxi/01.14 NCJD034-P/2015 H5N6 NP
EPI ISL 219874 A/Dk/Taiwan/A3400/2015 H5N8 NP
EPI ISL 231685 A/black-headed gull/Tyva/41/2016 H5N8 NP
EPI ISL 256462 A/M swan/Hungary/5879/2017 H5N5 NP
EPI ISL 240110 A/Ch/Astrakhan/3131/2016 H5N8 NP
EPI ISL 254745 A/Mal Dk/Korea/WA137/2017 H5N8 NP
EPI ISL 237554 A/painted stork/India/10CA03/2016 H5N8 NP
EPI ISL 234400 A/Mal/Poland/266/2010 H5N6 NP
EPI ISL 179386 A/Dk/Quang Ninh/19c511/2013 H5N8 NP
EPI ISL 200277 A/Dk/Hubei/03.06 WHWTZ0150-P/2015 H5N6 NP
EPI ISL 243050 A/barnacle Go/Germany-SH/R11505/2016 H5N5 NP
EPI ISL 259525 A/egret/Germany-SH/R1459/2017 H5N5 NP
EPI ISL 243049 A/Ch/Germany-SH/R425/2017 H5N5 NP
EPI ISL 249681 A/Ch/Germany-BB/R234ff/2017 H5N8 NP
EPI ISL 240012 A/Dk/France/161108h/2016 H5N8 NP
EPI ISL 237553 A/Dk/India/10CA01/2016 H5N8 NP
LC335984.1-A/M swan/Shimane/3211A001/2017-H5N6 NP
EPI ISL 287800 A/spoonbill/Taiwan/DB645/2017 H5N6 NP
EPI ISL 288364 A/Ch/Greece/39 2017b/2017 H5N6 NP
EPI ISL 238037 A/Ch/Germany-SH/R8595/2016 H5N8 NP
EPI ISL 274858 A/M swan/Kaliningrad/132/2017 H5N8-NP
EPI ISL 287564 A/T Dk/NL-Werkendam/16014159-001/2016 H5N5-NP
EPI ISL 256298 A/gadwall/Kurgan/2442/2016 H5N8-NP
EPI ISL 247724 A/wild Dk/Tatarstan/3059/2016 H5N8-NP
EPI ISL 288410 A/M swan/NL-Elburg/17017377-001/2017 H5N6 NP
EPI ISL 288412 A/T-Dk/NL-Hulshorst/17017367-007/2017 H5N6 NP
EPI ISL 288409 A/M Swan/NL-Hulshorst/17017367-012/2017 H5N6 NP
EPI ISL 287907 A/Dk/NL Biddinghuizen/17017237-001005/2017-H5N6 NP
EPI ISL 1108 A/Dk/Potsdam/2216-4/1984 H5N6 NP
EPI ISL 5884 A/Ch/Ireland/1378/1983 H5N8 NP
N.American LPAI H5 viruses
0.01

F

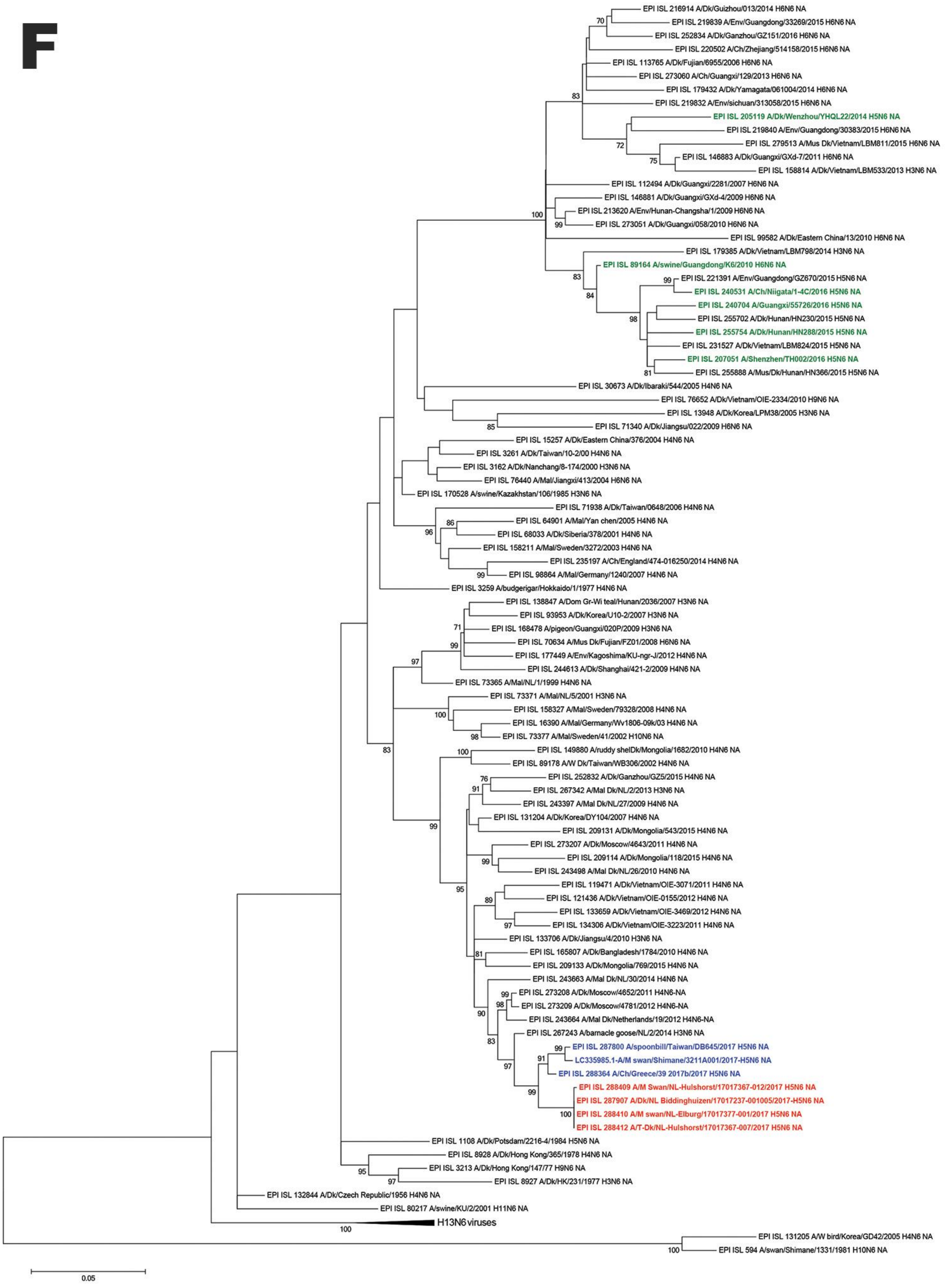
EPI ISL 216914 A/Dk/Guizhou/013/2014 H6N6 NA
EPI ISL 219839 A/Env/Guangdong/33269/2015 H6N6 NA
EPI ISL 252834 A/Dk/Ganzhou/GZ151/2016 H6N6 NA
EPI ISL 220502 A/Ch/Zhejiang/514158/2015 H6N6 NA
EPI ISL 113765 A/Dk/Fujian/6955/2006 H6N6 NA
EPI ISL 273060 A/Ch/Guangxi/129/2013 H6N6 NA
EPI ISL 179432 A/Dk/Yamagata/061004/2014 H6N6 NA
EPI ISL 219832 A/Env/sichuan/313058/2015 H6N6 NA
EPI ISL 205119 A/Dk/Wenzhou/YHQL22/2014 H5N6 NA
EPI ISL 219840 A/Env/Guangdong/30383/2015 H6N6 NA
EPI ISL 279513 A/Mus Dk/Vietnam/LBM811/2015 H6N6 NA
EPI ISL 146883 A/Dk/Guangxi/GXd-7/2011 H6N6 NA
EPI ISL 158814 A/Dk/Vietnam/LBM533/2013 H3N6 NA
EPI ISL 112494 A/Dk/Guangxi/2281/2007 H6N6 NA
EPI ISL 146881 A/Dk/Guangxi/GXd-4/2009 H6N6 NA
EPI ISL 213620 A/Env/Hunan-Changsha/1/2009 H6N6 NA
EPI ISL 273051 A/Dk/Guangxi/058/2010 H6N6 NA
EPI ISL 99582 A/Dk/Eastern China/13/2010 H6N6 NA
EPI ISL 179385 A/Dk/Vietnam/LBM798/2014 H3N6 NA
EPI ISL 89164 A/swine/Guangdong/K6/2010 H6N6 NA
EPI ISL 221391 A/Env/Guangdong/GZ670/2015 H5N6 NA
EPI ISL 240531 A/Ch/Niigata/1-4C/2016 H5N6 NA
EPI ISL 240704 A/Guangxi/55726/2016 H5N6 NA
EPI ISL 255702 A/Dk/Hunan/HN230/2015 H5N6 NA
EPI ISL 255754 A/Dk/Hunan/HN288/2015 H5N6 NA
EPI ISL 231527 A/Dk/Vietnam/LBM824/2015 H5N6 NA
EPI ISL 207051 A/Shenzhen/TH002/2016 H5N6 NA
EPI ISL 255888 A/Mus/Dk/Hunan/HN366/2015 H5N6 NA
EPI ISL 30673 A/Dk/Ibaraki/544/2005 H4N6 NA
EPI ISL 76652 A/Dk/Vietnam/OIE-2334/2010 H9N6 NA
EPI ISL 13948 A/Dk/Korea/LPM38/2005 H3N6 NA
EPI ISL 71340 A/Dk/Jiangsu/022/2009 H6N6 NA
EPI ISL 15257 A/Dk/Eastern China/376/2004 H4N6 NA
EPI ISL 3261 A/Dk/Taiwan/10-2/00 H4N6 NA
EPI ISL 3162 A/Dk/Nanchang/8-174/2000 H3N6 NA
EPI ISL 76440 A/Mal/Jiangxi/413/2004 H6N6 NA
EPI ISL 170528 A/swine/Kazakhstan/106/1985 H3N6 NA
EPI ISL 71938 A/Dk/Taiwan/0648/2006 H4N6 NA
EPI ISL 64901 A/Mal/Yan chen/2005 H4N6 NA
EPI ISL 68033 A/Dk/Siberia/378/2001 H4N6 NA
EPI ISL 158211 A/Mal/Sweden/3272/2003 H4N6 NA
EPI ISL 235197 A/Ch/England/474-016250/2014 H4N6 NA
EPI ISL 98864 A/Mal/Germany/1240/2007 H4N6 NA
EPI ISL 3259 A/budgerigar/Hokkaido/1/1977 H4N6 NA
EPI ISL 138847 A/Dom Gr-Wi teal/Hunan/2036/2007 H3N6 NA
EPI ISL 93953 A/Dk/Korea/U10-2/2007 H3N6 NA
EPI ISL 168478 A/pigeon/Guangxi/020P/2009 H3N6 NA
EPI ISL 70634 A/Mus Dk/Fujian/FZ01/2008 H6N6 NA
EPI ISL 177449 A/Env/Kagoshima/KU-ngr-J/2012 H4N6 NA
EPI ISL 244613 A/Dk/Shanghai/421-2/2009 H4N6 NA
EPI ISL 73365 A/Mal/NL/1/1999 H4N6 NA
EPI ISL 73371 A/Mal/NL/5/2001 H3N6 NA
EPI ISL 158327 A/Mal/Sweden/79328/2008 H4N6 NA
EPI ISL 16390 A/Mal/Germany/Wv1806-09k/03 H4N6 NA
EPI ISL 73377 A/Mal/Sweden/41/2002 H10N6 NA
EPI ISL 149880 A/ruddy shelDk/Mongolia/1682/2010 H4N6 NA
EPI ISL 89178 A/W Dk/Taiwan/WB306/2002 H4N6 NA
EPI ISL 252832 A/Dk/Ganzhou/GZ5/2015 H4N6 NA
EPI ISL 267342 A/Mal Dk/NL/2/2013 H3N6 NA
EPI ISL 243397 A/Mal Dk/NL/27/2009 H4N6 NA
EPI ISL 131204 A/Dk/Korea/DY104/2007 H4N6 NA
EPI ISL 209131 A/Dk/Mongolia/543/2015 H4N6 NA
EPI ISL 273207 A/Dk/Moscow/4643/2011 H4N6 NA
EPI ISL 209114 A/Dk/Mongolia/118/2015 H4N6 NA
EPI ISL 243498 A/Mal Dk/NL/26/2010 H4N6 NA
EPI ISL 119471 A/Dk/Vietnam/OIE-3071/2011 H4N6 NA
EPI ISL 121436 A/Dk/Vietnam/OIE-0155/2012 H4N6 NA
EPI ISL 133659 A/Dk/Vietnam/OIE-3469/2012 H4N6 NA
EPI ISL 134306 A/Dk/Vietnam/OIE-3223/2011 H4N6 NA
EPI ISL 133706 A/Dk/Jiangsu/4/2010 H3N6 NA
EPI ISL 165807 A/Dk/Bangladesh/1784/2010 H4N6 NA
EPI ISL 209133 A/Dk/Mongolia/769/2015 H4N6 NA
EPI ISL 243663 A/Mal Dk/NL/30/2014 H4N6 NA
EPI ISL 273208 A/Dk/Moscow/4652/2011 H4N6-NA
EPI ISL 273209 A/Dk/Moscow/4781/2012 H4N6-NA
EPI ISL 243664 A/Mal Dk/Netherlands/19/2012 H4N6-NA
EPI ISL 267243 A/barnacle goose/NL/2/2014 H3N6 NA
EPI ISL 287800 A/spoonbill/Taiwan/DB645/2017 H5N6 NA
LC335985.1-A/M swan/Shimane/3211A001/2017-H5N6 NA
EPI ISL 288364 A/Ch/Greece/39 2017b/2017 H5N6 NA
EPI ISL 288409 A/M Swan/NL-Hulshorst/17017367-012/2017 H5N6 NA
EPI ISL 287907 A/Dk/NL Biddinghuizen/17017237-001005/2017-H5N6 NA
EPI ISL 288410 A/M swan/NL-Elburg/17017377-001/2017 H5N6 NA
EPI ISL 288412 A/T-Dk/NL-Hulshorst/17017367-007/2017 H5N6 NA
EPI ISL 1108 A/Dk/Potsdam/2216-4/1984 H5N6 NA
EPI ISL 8928 A/Dk/Hong Kong/365/1978 H4N6 NA
EPI ISL 3213 A/Dk/Hong Kong/147/77 H9N6 NA
EPI ISL 8927 A/Dk/HK/231/1977 H3N6 NA
EPI ISL 132844 A/Dk/Czech Republic/1956 H4N6 NA
EPI ISL 80217 A/swine/KU/2/2001 H11N6 NA
H13N6 viruses
EPI ISL 131205 A/W bird/Korea/GD42/2005 H4N6 NA
EPI ISL 594 A/swan/Shimane/1331/1981 H10N6 NA
0.05

G

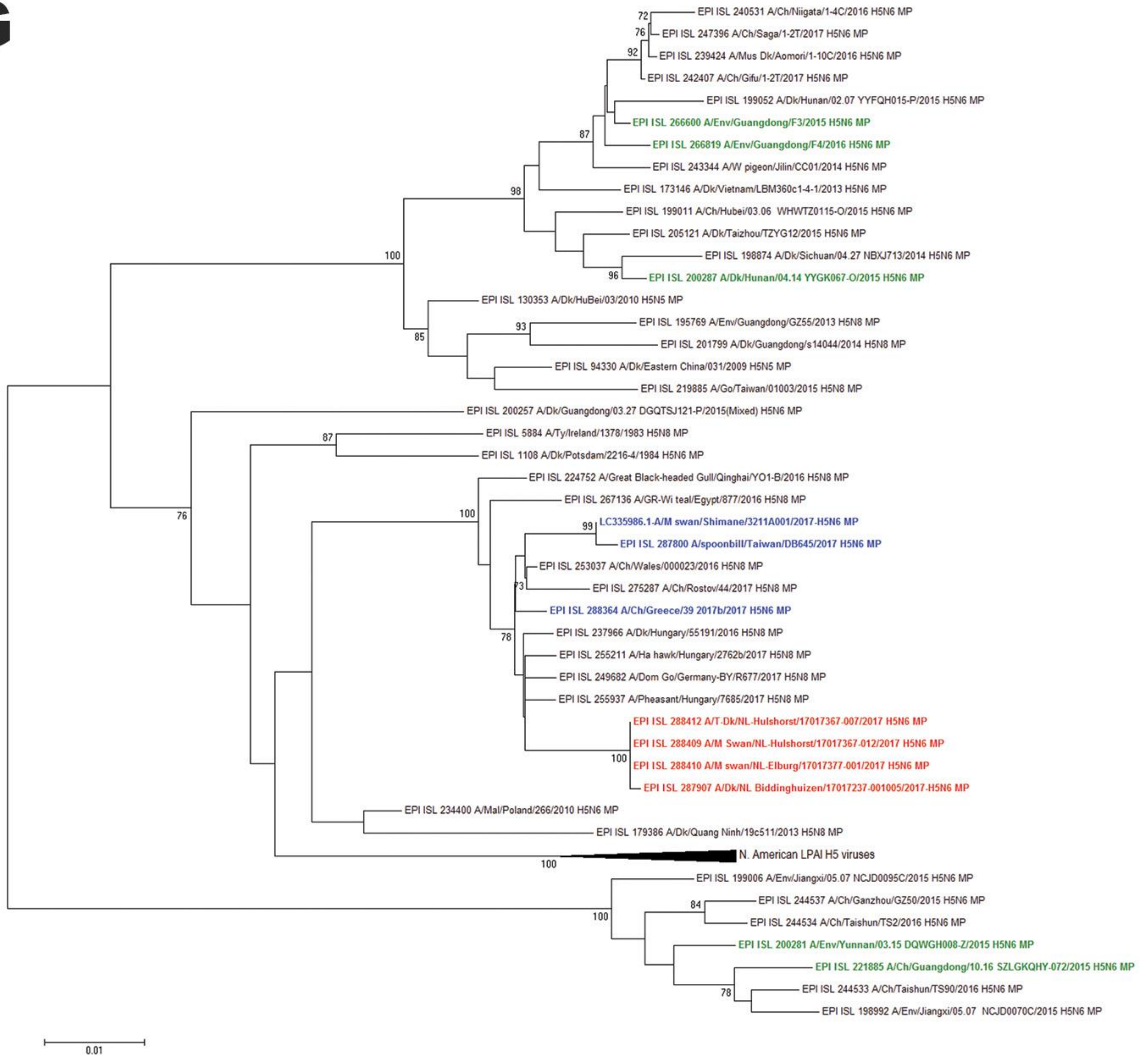

72
EPI ISL 240531 A/Ch/Niigata/1-4C/2016 H5N6 MP
76
EPI ISL 247396 A/Ch/Saga/1-2T/2017 H5N6 MP
92
EPI ISL 239424 A/Mus Dk/Aomori/1-10C/2016 H5N6 MP
EPI ISL 242407 A/Ch/Gifu/1-2T/2017 H5N6 MP
EPI ISL 199052 A/Dk/Hunan/02.07 YYFQH015-P/2015 H5N6 MP
EPI ISL 266600 A/Env/Guangdong/F3/2015 H5N6 MP
87
EPI ISL 266819 A/Env/Guangdong/F4/2016 H5N6 MP
EPI ISL 243344 A/W pigeon/Jilin/CC01/2014 H5N6 MP
98
EPI ISL 173146 A/Dk/Vietnam/LBM360c1-4-1/2013 H5N6 MP
EPI ISL 199011 A/Ch/Hubei/03.06 WHWTZ0115-O/2015 H5N6 MP
EPI ISL 205121 A/Dk/Taizhou/TZYG12/2015 H5N6 MP
100
EPI ISL 198874 A/Dk/Sichuan/04.27 NBXJ713/2014 H5N6 MP
96
EPI ISL 200287 A/Dk/Hunan/04.14 YYGK067-O/2015 H5N6 MP
EPI ISL 130353 A/Dk/HuBei/03/2010 H5N5 MP
93
EPI ISL 195769 A/Env/Guangdong/GZ55/2013 H5N8 MP
85
EPI ISL 201799 A/Dk/Guangdong/s14044/2014 H5N8 MP
EPI ISL 94330 A/Dk/Eastern China/031/2009 H5N5 MP
EPI ISL 219885 A/Go/Taiwan/01003/2015 H5N8 MP
EPI ISL 200257 A/Dk/Guangdong/03.27 DGQTSJ121-P/2015(Mixed) H5N6 MP
87
EPI ISL 5884 A/Ty/Ireland/1378/1983 H5N8 MP
EPI ISL 1108 A/Dk/Potsdam/2216-4/1984 H5N6 MP
EPI ISL 224752 A/Great Black-headed Gull/Qinghai/YO1-B/2016 H5N8 MP
EPI ISL 267136 A/GR-Wi teal/Egypt/877/2016 H5N8 MP
100
76
99
LC335986.1-A/M swan/Shimane/3211A001/2017-H5N6 MP
EPI ISL 287800 A/spoonbill/Taiwan/DB645/2017 H5N6 MP
EPI ISL 253037 A/Ch/Wales/000023/2016 H5N8 MP
73
EPI ISL 275287 A/Ch/Rostov/44/2017 H5N8 MP
EPI ISL 288364 A/Ch/Greece/39 2017b/2017 H5N6 MP
78
EPI ISL 237966 A/Dk/Hungary/55191/2016 H5N8 MP
EPI ISL 255211 A/Ha hawk/Hungary/2762b/2017 H5N8 MP
EPI ISL 249682 A/Dom Go/Germany-BY/R677/2017 H5N8 MP
EPI ISL 255937 A/Pheasant/Hungary/7685/2017 H5N8 MP
EPI ISL 288412 A/T-Dk/NL-Hulshorst/17017367-007/2017 H5N6 MP
EPI ISL 288409 A/M Swan/NL-Hulshorst/17017367-012/2017 H5N6 MP
100
EPI ISL 288410 A/M swan/NL-Elburg/17017377-001/2017 H5N6 MP
EPI ISL 287907 A/Dk/NL Biddinghuizen/17017237-001005/2017-H5N6 MP
EPI ISL 234400 A/Mal/Poland/266/2010 H5N6 MP
EPI ISL 179386 A/Dk/Quang Ninh/19c511/2013 H5N8 MP
N. American LPAI H5 viruses
100
EPI ISL 199006 A/Env/Jiangxi/05.07 NCJD0095C/2015 H5N6 MP
84
EPI ISL 244537 A/Ch/Ganzhou/GZ50/2015 H5N6 MP
100
EPI ISL 244534 A/Ch/Taishun/TS2/2016 H5N6 MP
EPI ISL 200281 A/Env/Yunnan/03.15 DQWGH008-Z/2015 H5N6 MP
EPI ISL 221885 A/Ch/Guangdong/10.16 SZLGKQHY-072/2015 H5N6 MP
78
EPI ISL 244533 A/Ch/Taishun/TS90/2016 H5N6 MP
EPI ISL 198992 A/Env/Jiangxi/05.07 NCJD0070C/2015 H5N6 MP
0.01

H

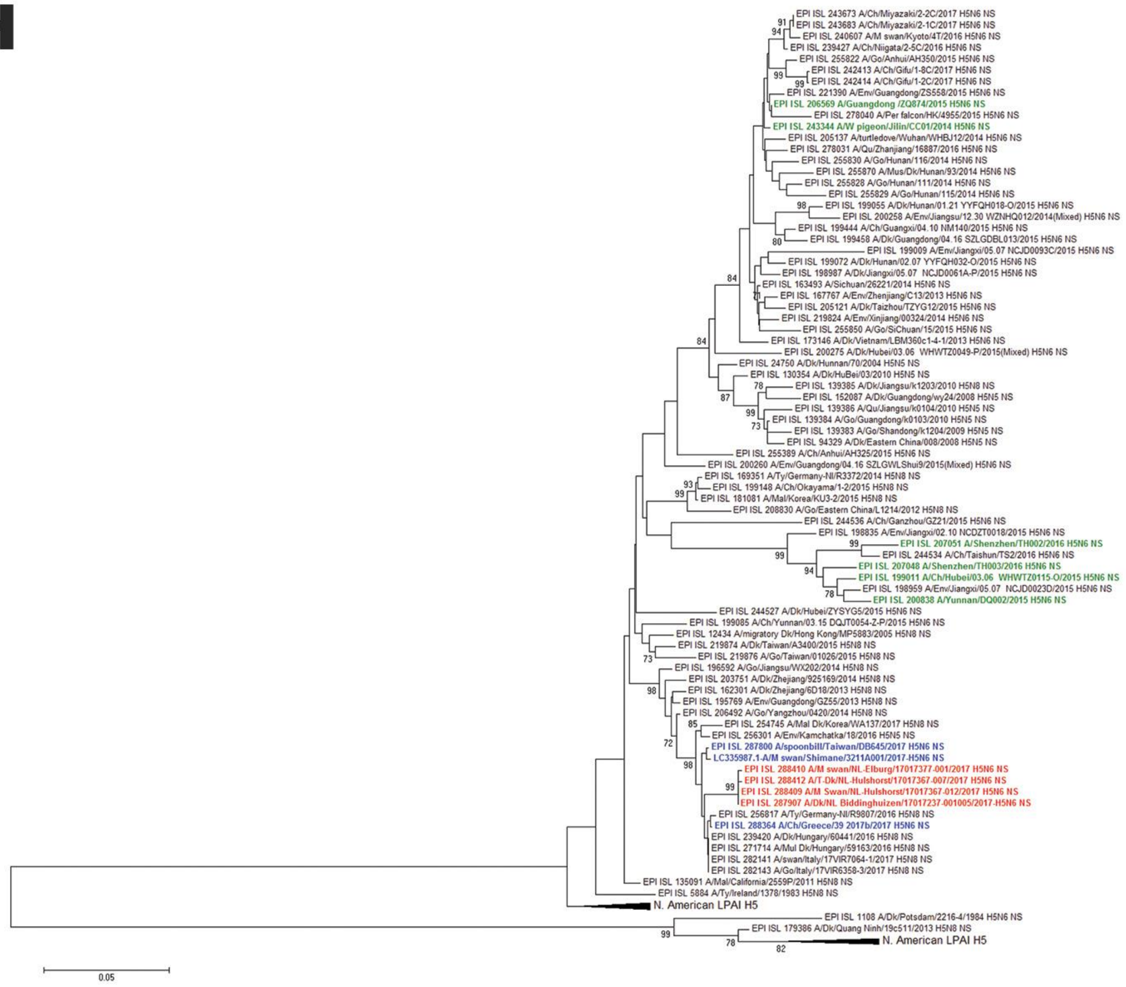

Technical Appendix Figure 2 (following pages). Phylogenetic analysis was performed for each gene segment. Related sequences in the GISAID database (http://platform.gisaid.org) were clustered using the CD-HIT algorithm, using identity setting of 0.97–0.985. Phylogenetic trees were generated using the neighbor-joining method with 1,000 bootstrap replicates within the MEGA6 software package. The maximum composite likelihood model was used with a gamma distribution (shape parameter = 1) for rate variation. The optimal phylogenetic trees are shown, and are drawn to scale. The GISAID accession numbers of the viruses are shown in the trees, as are the novel viruses isolated from the commercial duck farm (EPI ISL 287907), the tufted (EPI ISL 288412), and the 2 mute swans (EPI ISL 288409 and EPI ISL 288410). Groups A–D of clade 2.3.4.4 are indicated. The H5N6 viruses isolated in the Netherlands are in red, the H5N6 viruses isolated in Greece, Japan, and Taiwan are in blue, and the taxons comprising H5N6 viruses isolated from humans are in green. Segments shown are A) polymerase basic (PB2); B) polymerase basic (PB1); C) polymerase (PA); D) hemagglutinin (HA); E) nucleoprotein (NP); F) neuraminidase (NA); G) matrix protein (MP); H) nonstructural protein (NS).

A

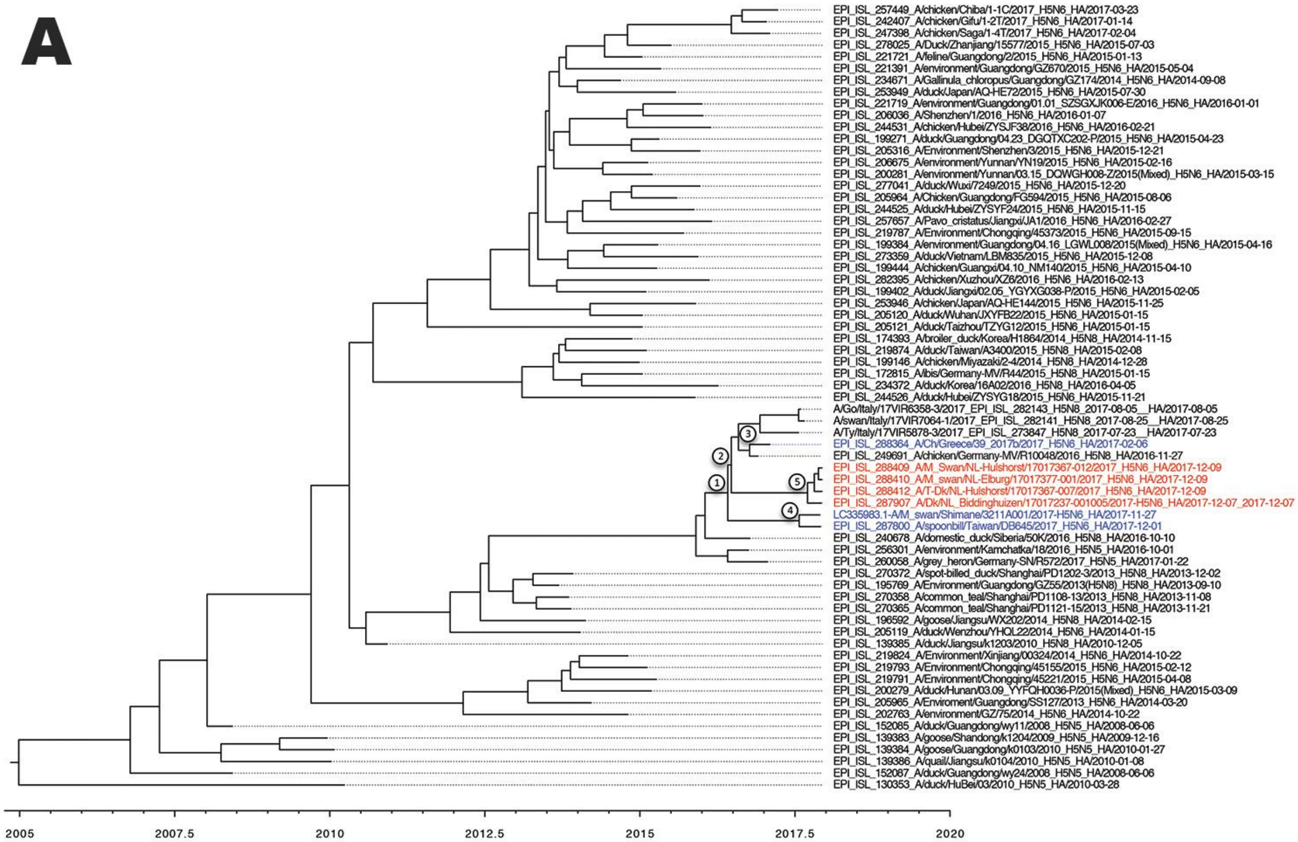
EPI_ISL_257449_A/chicken/Chiba/1-1C/2017_H5N6_HA/2017-03-23
EPI_ISL_242407_A/chicken/Gifu/1-2T/2017_H5N6_HA/2017-01-14
EPI_ISL_247398_A/chicken/Saga/1-4T/2017_H5N6_HA/2017-02-04
EPI_ISL_278025_A/Duck/Zhanjiang/15577/2015_H5N6_HA/2015-07-03
EPI_ISL_221721_A/feline/Guangdong/2/2015_H5N6_HA/2015-01-13
EPI_ISL_221391_A/environment/Guangdong/GZ670/2015_H5N6_HA/2015-05-04
EPI_ISL_234671_A/Gallinula_chloropus/Guangdong/GZ174/2014_H5N6_HA/2014-09-08
EPI_ISL_253949_A/duck/Japan/AQ-HE72/2015_H5N6_HA/2015-07-30
EPI_ISL_221719_A/environment/Guangdong/01.01_SZSGXJK006-E/2016_H5N6_HA/2016-01-01
EPI_ISL_206036_A/Shenzhen/1/2016_H5N6_HA/2016-01-07
EPI_ISL_244531_A/chicken/Hubei/ZYSJF38/2016_H5N6_HA/2016-02-21
EPI_ISL_199271_A/duck/Guangdong/04.23_DGQTXC202-P/2015_H5N6_HA/2015-04-23
EPI_ISL_205316_A/Environment/Shenzhen/3/2015_H5N6_HA/2015-12-21
EPI_ISL_206675_A/environment/Yunnan/YN19/2015_H5N6_HA/2015-02-16
EPI_ISL_200281_A/environment/Yunnan/03.15_DQWGH008-Z/2015(Mixed)_H5N6_HA/2015-03-15
EPI_ISL_277041_A/duck/Wuxi/7249/2015_H5N6_HA/2015-12-20
EPI_ISL_205964_A/Chicken/Guangdong/FG594/2015_H5N6_HA/2015-08-06
EPI_ISL_244525_A/duck/Hubei/ZYSYF24/2015_H5N6_HA/2015-11-15
EPI_ISL_257657_A/Pavo_cristatus/Jiangxi/JA1/2016_H5N6_HA/2016-02-27
EPI_ISL_219787_A/Environment/Chongqing/45373/2015_H5N6_HA/2015-09-15
EPI_ISL_199384_A/environment/Guangdong/04.16_LGWL008/2015(Mixed)_H5N6_HA/2015-04-16
EPI_ISL_273359_A/duck/Vietnam/LBM835/2015_H5N6_HA/2015-12-08
EPI_ISL_199444_A/chicken/Guangxi/04.10_NM140/2015_H5N6_HA/2015-04-10
EPI_ISL_282395_A/chicken/Xuzhou/XZ6/2016_H5N6_HA/2016-02-13
EPI_ISL_199402_A/duck/Jiangxi/02.05_YGYXG038-P/2015_H5N6_HA/2015-02-05
EPI_ISL_253946_A/chicken/Japan/AQ-HE144/2015_H5N6_HA/2015-11-25
EPI_ISL_205120_A/duck/Wuhan/JXYFB22/2015_H5N6_HA/2015-01-15
EPI_ISL_205121_A/duck/Taizhou/TZYG12/2015_H5N6_HA/2015-01-15
EPI_ISL_174393_A/broiler_duck/Korea/H1864/2014_H5N8_HA/2014-11-15
EPI_ISL_219874_A/duck/Taiwan/A3400/2015_H5N8_HA/2015-02-08
EPI_ISL_199146_A/chicken/Miyazaki/2-4/2014_H5N8_HA/2014-12-28
EPI_ISL_172815_A/ibis/Germany-MV/R44/2015_H5N8_HA/2015-01-15
EPI_ISL_234372_A/duck/Korea/16A02/2016_H5N8_HA/2016-04-05
EPI_ISL_244526_A/duck/Hubei/ZYSYG18/2015_H5N6_HA/2015-11-21
A/Go/Italy/17VIR6358-3/2017_EPI_ISL_282143_H5N8_2017-08-05__HA/2017-08-05
A/swan/Italy/17VIR7064-1/2017_EPI_ISL_282141_H5N8_2017-08-25__HA/2017-08-25
A/Ty/Italy/17VIR5878-3/2017_EPI_ISL_273847_H5N8_2017-07-23__HA/2017-07-23
EPI_ISL_288364_A/Ch/Greece/39_2017b/2017_H5N6_HA/2017-02-06
EPI_ISL_249691_A/chicken/Germany-MV/R10048/2016_H5N8_HA/2016-11-27
EPI_ISL_288409_A/M_Swan/NL-Hulshorst/17017367-012/2017_H5N6_HA/2017-12-09
EPI_ISL_288410_A/M_swan/NL-Elburg/17017377-001/2017_H5N6_HA/2017-12-09
EPI_ISL_288412_A/T-Dk/NL-Hulshorst/17017367-007/2017_H5N6_HA/2017-12-09
EPI_ISL_287907_A/Dk/NL_Biddinghuizen/17017237-001005/2017-H5N6_HA/2017-12-07_2017-12-07
LC335983.1-A/M_swan/Shimane/3211A001/2017-H5N6_HA/2017-11-27
EPI_ISL_287800_A/spoonbill/Taiwan/DB645/2017_H5N6_HA/2017-12-01
EPI_ISL_240678_A/domestic_duck/Siberia/50K/2016_H5N8_HA/2016-10-10
EPI_ISL_256301_A/environment/Kamchatka/18/2016_H5N5_HA/2016-10-01
EPI_ISL_260058_A/grey_heron/Germany-SN/R572/2017_H5N5_HA/2017-01-22
EPI_ISL_270372_A/spot-billed_duck/Shanghai/PD1202-3/2013_H5N8_HA/2013-12-02
EPI_ISL_195769_A/Environment/Guangdong/GZ55/2013(H5N8)_H5N8_HA/2013-09-10
EPI_ISL_270358_A/common_teal/Shanghai/PD1108-13/2013_H5N8_HA/2013-11-08
EPI_ISL_270365_A/common_teal/Shanghai/PD1121-15/2013_H5N8_HA/2013-11-21
EPI_ISL_196592_A/goose/Jiangsu/WX202/2014_H5N8_HA/2014-02-15
EPI_ISL_205119_A/duck/Wenzhou/YHQL22/2014_H5N6_HA/2014-01-15
EPI_ISL_139385_A/duck/Jiangsu/k1203/2010_H5N8_HA/2010-12-05
EPI_ISL_219824_A/Environment/Xinjiang/00324/2014_H5N6_HA/2014-10-22
EPI_ISL_219793_A/Environment/Chongqing/45155/2015_H5N6_HA/2015-02-12
EPI_ISL_219791_A/Environment/Chongqing/45221/2015_H5N6_HA/2015-04-08
EPI_ISL_200279_A/duck/Hunan/03.09_YYFQH0036-P/2015(Mixed)_H5N6_HA/2015-03-09
EPI_ISL_205965_A/Enviroment/Guangdong/SS127/2013_H5N6_HA/2014-03-20
EPI_ISL_202763_A/environment/GZ/75/2014_H5N6_HA/2014-10-22
EPI_ISL_152085_A/duck/Guangdong/wy11/2008_H5N5_HA/2008-06-06
EPI_ISL_139383_A/goose/Shandong/k1204/2009_H5N5_HA/2009-12-16
EPI_ISL_139384_A/goose/Guangdong/k0103/2010_H5N5_HA/2010-01-27
EPI_ISL_139386_A/quail/Jiangsu/k0104/2010_H5N5_HA/2010-01-08
EPI_ISL_152087_A/duck/Guangdong/wy24/2008_H5N5_HA/2008-06-06
EPI_ISL_130353_A/duck/HuBei/03/2010_H5N5_HA/2010-03-28
1
2
3
4
5
2005
2007.5
2010
2012.5
2015
2017.5
2020

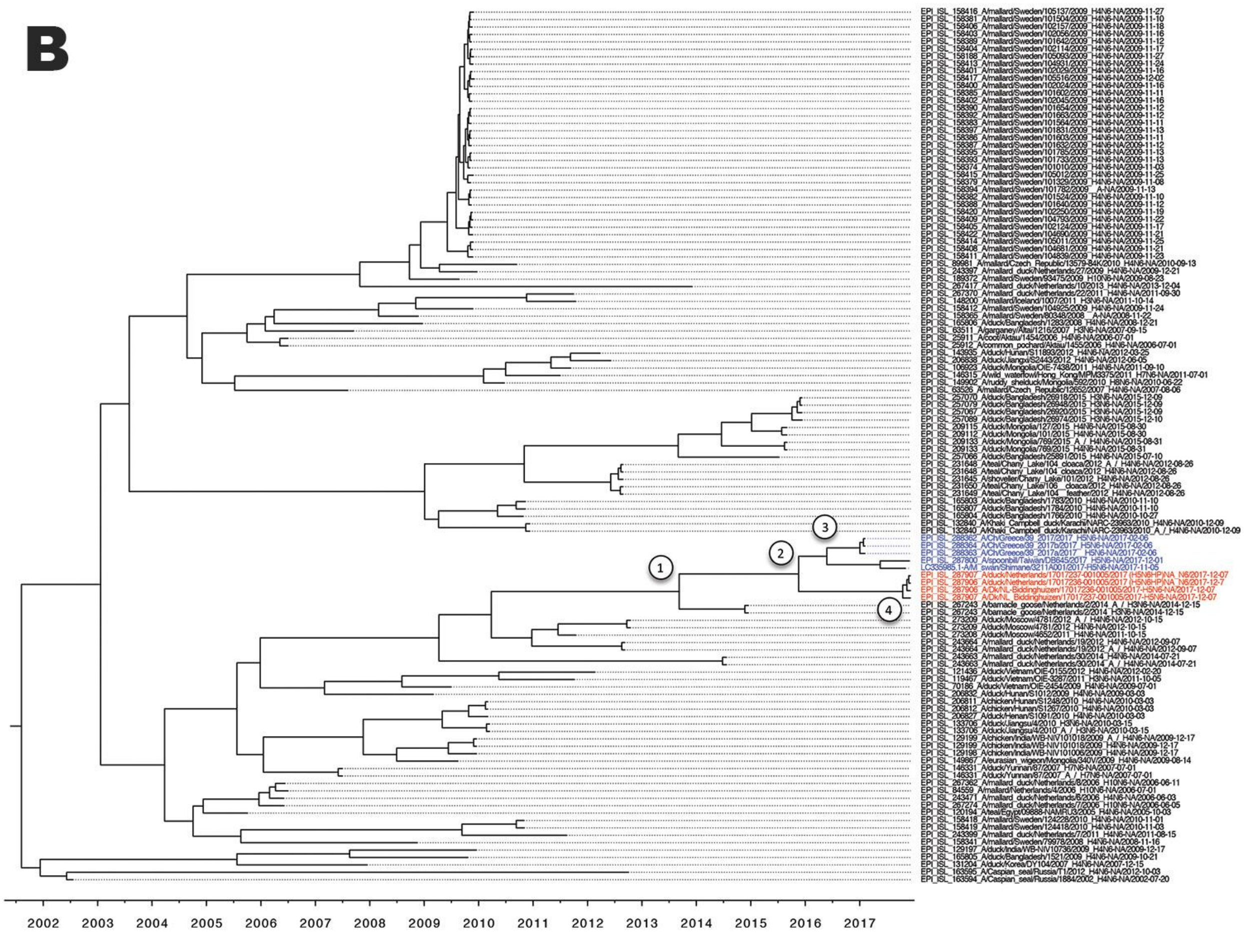

Technical Appendix Figure 3 (following pages). Molecular dating was performed for the (A) hemagglutinin (HA) and (B) neuraminidase (NA) gene segments. We estimated the tMRCA of 86 HA and 118 NA gene segment sequences; the GISAID accession numbers of the viruses are shown in the trees. Time scaled phylogenies (dates shown on the horizontal axis) were reconstructed using a Bayesian Markov chain Monte Carlo (MCMC) framework implemented in the BEAST software package (v 1.8.4) (http://beast.community/beast) with the SRD06 nt substitution model, the Bayesian Skyline coalescent model, and an uncorrelated lognormal relaxed molecular clock. Three MCMC runs of 50 × 106 states sampling each 5,000 steps were combined to obtain an effective sample size of >200. Maximum clade credibility (MCC) trees were reconstructed with 10% burn-in and the posterior distribution of relevant parameters were assessed in Fig Tree v 1.4.3 (http://tree.bio.ed.ac.uk/software/figtree). The H5N6 viruses isolated in the Netherlands are in red and those isolated in Greece, Japan, and Taiwan are in blue.
